# Antimicrobial potentiality of actinobacteria isolated from two microbiologically unexplored forest ecosystems of Northeast India

**DOI:** 10.1186/s12866-018-1215-7

**Published:** 2018-07-11

**Authors:** Ranjita Das, Wahengbam Romi, Rictika Das, Hridip Kumar Sharma, Debajit Thakur

**Affiliations:** 1 Microbial Biotechnology Laboratory, Life Sciences Division, Institute of Advanced Study in Science and Technology (IASST), Paschim Boragaon, Garchuk, Guwahati, Assam 781035 India; 2 Molecular Biology and Microbial Biotechnology Laboratory, Life Sciences Division, Institute of Advanced Study in Science and Technology (IASST), Guwahati, Assam India; 3 Department of Biotechnology, Gauhati University, Guwahati, Assam India

**Keywords:** Forest ecosystem, Actinobacteria, *Streptomyces* sp., Antimicrobial activity, Antimicrobial biosynthetic gene, MRSA

## Abstract

**Background:**

Actinobacteria are often known to be great producers of antibiotics. The rapid increase in the global burden of antibiotic-resistance with the concurrent decline in the discovery of new antimicrobial molecules necessitates the search for novel and effective antimicrobial metabolites from unexplored ecological niches. The present study investigated the antimicrobial producing actinobacterial strains isolated from the soils of two microbiologically unexplored forest ecosystems, viz. Nameri National Park (NNP) and Panidehing Wildlife Sanctuary (PWS), located in the Eastern Himalayan Biodiversity hotspot region.

**Results:**

A total of 172 putative isolates of actinobacteria were isolated, of which 24 isolates showed strong antimicrobial bioactivity. Evaluation of the ethyl acetate extracts of culture supernatants against test microbial strains revealed that isolates PWS22, PWS41, PWS12, PWS52, PWS11, NNPR15, NNPR38, and NNPR69 were the potent producers of antimicrobial metabolites. The antimicrobial isolates dominantly belonged to *Streptomyces*, followed by *Nocardia* and *Streptosporangium*. Some of these isolates could be putative novel taxa. Analysis of the antimicrobial biosynthetic genes (type II polyketide synthase and nonribosomal peptide synthetase genes) showed that the antimicrobial metabolites were associated with pigment production and belonged to known families of bioactive secondary metabolites. Characterization of the antimicrobial metabolites of *Streptomyces* sp. PWS52, which showed lowest taxonomic identity among the studied potent antimicrobial metabolite producers, and their interaction with the test strains using GC-MS, UHPLC-MS, and scanning electron microscopy revealed that the potential bioactivity of PWS52 was due to the production of active antifungal and antibacterial metabolites like 2,5-bis(1,1-dimethylethyl) phenol, benzeneacetic acid and nalidixic acid.

**Conclusions:**

Our findings suggest that the unexplored soil habitats of NNP and PWS forest ecosystems of Northeast India harbor previously undescribed actinobacteria with the capability to produce diverse antimicrobial metabolites that may be explored to overcome the rapidly rising global concern about antibiotic-resistance.

**Electronic supplementary material:**

The online version of this article (10.1186/s12866-018-1215-7) contains supplementary material, which is available to authorized users.

## Background

Over 1 million natural compounds are available in the world, among which 5% are of microbial origin [1]. Microbial-derived natural compounds are the major sources of persistently used antibiotics today [2, 3], and many active microbial strains have made an enormous contribution in the field of medical biology towards drug discovery and development process [1]. However, due to less and improper pharmaceutical knowledge among the common people, antibiotics are inappropriately used over a prolonged period, which result in resistance among the pathogenic microorganisms. Such microorganisms develop antibiotic resistance by acquiring different resistant-causing genes in their genomes through horizontal gene transfer, which finally results in the development of various mechanisms to inactivate antibiotics [4]. Thus, there is a need for new antibiotics and bioactive metabolites to target the emerging multidrug-resistant microbial pathogens that cause life-threatening infections.

Soil contains diverse ecological niches, the inhabitants of which produce various biologically active natural compounds including antibiotics that have clinical significance. Actinobacteria represent a significant proportion of most of the soil microbial population including the forest soil [5]. Actinobacteria are Gram-positive bacteria that constitute one of the largest bacterial phyla with high G + C DNA. Actinobacteria have diverse physiological effects that make them the dominant performers in biotechnology industry for the production of natural bioactive metabolites like enzyme inhibitors, immunomodifiers, antibiotics, plant growth promoting substances, natural dyes and other compounds of biotechnological interest [6, 7]. The importance of these organisms is clearly seen from the fact that over 5000 compounds have been reported from actinobacteria that contributed to the development of 90% of the commercial antibiotics being used for either clinical or research needs [8]. Members of *Actinobacteriaceae* family are known producers of polyketides and nonribosomal polyketide peptides by polyketide pathways (type-I and type-II) and nonribosomal peptide synthetase pathways, respectively, which are the dominant pathways for the production of secondary metabolites in this group of bacteria [9].

*Streptomyces* constitutes the largest genus of actinobacteria with 826 species [10]. The species of this genus are the most vital sources of natural compounds with a pragmatic record of producing novel bioactive molecules, including several commercially important drugs like ivermectin, tetracycline, streptomycin, nystatin, etc. [11]. During the past years, several antibacterial, antifungal, antioxidant and antitumor compounds were purified and characterized from *Streptomyces* spp. [5, 12–16]. Many research groups have been trying to explore novel actinobacterial strains from different terrestrial habitats to find new drug molecules against various diseases. Besides that, less fraction of the culturable actinobacteria have been recovered until today [17]. Also, the likelihood of discovering novel antimicrobial metabolites from these distinctive species has recently decreased as culture extracts have frequently yielded a high number of known compounds [12, 18]. Shifting the search away from explored environments to unexplored ones could increase the possibility of discovering or formulating new molecules [19]. Unexplored and underexplored areas include biodiversity hotspots, the unique environment of which affects the species diversity and may result in the evolution of secondary metabolite pathways [20]. Northeast India falls under the Eastern Himalayan and the Indo-Burma mega-biodiversity hotspots that have diverse forest ecosystems such as wetland, tropical and temperate rainforests [21]. The protected area network in Assam, a state in Northeast India, occupies an area of 3925 km^2^ and constitutes about 5% of the state’s geographical area [22], which plays a very important role in the in situ conservation of biodiversity. Various investigations focused on the exploration of actinobacterial isolates from the protected area ecosystems of Northeast India for antimicrobial activities have been reported [19, 23–26]. However, many natural forest ecosystems of Assam are still either unexplored or underexplored, and thus are productive resources for the isolation of lesser-studied microorganisms including actinobacteria.

The present study aimed to investigate actinobacterial species with antimicrobial potential in the soils of two microbiologically unexplored forest ecosystems located in the Eastern Himalayan Biodiversity hotspot. With a contrasting environment existing in the ecosystems, the study hypothesizes that these pristine natural habitats harbor actinobacterial species possessing unique and efficient antimicrobial capability. We isolated soil actinobacteria prevalent in these locations, evaluated the phylogenetic relatedness among the potent antimicrobial metabolite producing isolates, detected the polyketide synthase type II (PKSII) and nonribosomal peptide synthetase (NRPS) genes, and performed metabolite analysis and interaction of the active extract of a potent antimicrobial isolate against test microbial strains.

## Methods

### Sampling sites and sample collection

The two forest ecosystems viz. Nameri National Park (NNP) (27°0′36′′N, 92°47′24′′E, covering 220 km^2^) and Panidehing Wildlife Sanctuary (PWS) (27°7′19′′N, 94°35′47′′E, covering 33.93 km^2^) are located in the Sonitpur and Sivasagar districts, respectively, of Assam, India. The NNP geographically comes under the Brahmaputra valley and experiences semi-evergreen deciduous forest, while the PWS is situated in the wetlands spread along the rivers Disang and Demow and covered with swampy soil. Soil samples were collected during March to May 2013. Seven different sites, each covering several diverse habitats, were randomly selected in both the forest ecosystems for collection of soil samples. These habitats included the forest tree rhizosphere, grassland, forest litter and swampy area. At each site, five replicate soil samples (each weighing ~100 g) were randomly collected at 5–20 cm depth below the surface within an area of 50 m^2^. The replicate samples were bulked and homogenized to prepare a composite sample, resulting in 14 composite soil samples. The collected soil samples were carried aseptically in sterile zip-lock bags, transported to the laboratory using frost packs and stored at 4 °C till further processing. Isolation of actinobacteria was completed within 5 days of sample collection. The study was conducted as per the “Guidelines on Access to Biological Resources and Associated Knowledge and Benefits Sharing Regulations” of the National Biodiversity Authority, Ministry of Environment, Forest and Climate Change, Government of India, and the permissions for the collection of soil samples from the protected forests were obtained from the concerned Protected Area Managers, Forest Department, Government of Assam, India.

### Isolation of actinobacteria

Selective isolation of actinobacteria was carried out by the serial dilution technique as described previously [19]. One gram of each composite soil sample was suspended in 100 ml of physiological saline (NaCl, 9 g/l) and incubated at 28 °C in constantly shaking condition at 200 rpm for 30 min. A 10^− 4^ dilution of the soil suspension was made using serial dilution procedure and 0.1 ml of each dilution was spread plated in duplicates onto four selective media: International *Streptomyces* Project Yeast Malt Agar medium (ISP2) (M424, HiMedia, India), *Streptomyces* Agar (SA) medium (M1352, HiMedia), Actinomycete Isolation Agar (AIA) medium (M490, HiMedia) and GLM agar medium (yeast extract, 3 g; malt extract, 3 g; peptone type-I, 5 g; starch, 10 g; agar, 20 g; distilled water, 1000 ml; pH 7.5) [23]. Isolation media were supplemented with rifampicin (25 μg/ml), nalidixic acid (25 μg/ml) and amphotericin B (75 μg/ml). The plates were incubated at 28 °C for 5−20 days and observed on a daily basis to check the colony growth. Based on colony morphology, different actinobacterial isolates were selected and purified by streaking in GLM medium. The purified putative actinobacterial isolates were subculture on GLM slants, incubated at 28 °C to achieve good sporulation and then preserved at 4 °C. For long-term storage, the cultures were preserved in 20% (*v*/v) glycerol at − 80 °C as a stock culture [27].

### Test microbial strains

*Staphylococcus aureus* MTCC 96, *Escherichia coli* MTCC 40 and *Candida albicans* MTCC 227 were purchased from Microbial Type Culture Collection, CSIR-Institute of Microbial Technology, Chandigarh, India, and the methicillin-resistant *Staphylococcus aureus* (MRSA) ATCC 43300 was purchased from HiMedia. Two clinical bacterial isolates (CL1 and CL2) were also obtained from Microbiology Department, Guwahati Neurological Research Center (GNRC), Guwahati, Assam for the evaluation of the antimicrobial assay. These two clinical isolates belonged to *Staphylococcus saprophyticus* and *Bacillus pumillus*, respectively, as determined by their 16S rRNA gene sequencing (date not shown). The antibiotic sensitivity pattern of the two clinical isolates was checked against 12 antibiotics using antibiotic-impregnated disc procured from HiMedia (data not shown). The test bacterial strains were cultured in Nutrient Agar (NA) medium (M001, HiMedia) at 37 °C while *C. albicans* was cultured in Sabouraud Dextrose Agar (SDA) medium (M063, HiMedia) at 28 °C. All the test microbial strains were preserved at − 80 °C in 20% (*v*/v) glycerol as stock cultures.

### Preliminary screening of antimicrobial activity

All the isolated cultures were checked for antimicrobial metabolite production by spot inoculation method [23]. The actinobacterial isolates were spot inoculated on AIA medium and incubated at 28 °C for 6 days. Freshly grown test microbial strains (MTCC 40, MTCC 96, MTCC 227) were inoculated into 20 ml Nutrient Broth (NB) (M002, HiMedia) (for bacteria) and Sabouraud Dextrose Broth (SDB) (ME033, HiMedia) (for fungus) and incubated in their respective conditions with shaking at 200 rpm to get a turbidity of 0.5 McFarland (10^8^ CFU/ml). The actinobacterial colonies were then laid over with semi-solid (0.6% *w*/*v* agar) SDA medium (for fungi) and NA medium (for bacteria) that were previously seeded with 10^8^ CFU of one of the test microbial strains. The antimicrobial activity, which was indicated by the zone of inhibition, was observed after 24 h incubation at 37 °C for bacteria and 28 °C for *C. albicans*.

### Production of antimicrobial metabolites in submerged culture

A 2 cm^2^ piece of substrate mycelia grown on AIA plate was scooped out from each 7-days old actinobacterial isolate that showed antimicrobial activity in the previous screening and inoculated separately in 50 ml GLM broth. The cultures were then grown in a rotary shaker at 28°C and 200 rpm for 7 days. The resulting culture broths were separated from the mycelia by centrifugation at 10000 rpm for 15 min. After filter sterilization, the cell-free culture supernatant was used for the screening of extracellular antimicrobial metabolite production against the test MTCC microbial strains by agar well diffusion method using Mueller Hinton Agar (MHA) medium (M173, HiMedia) that was previously seeded with 10^8^ CFU of one of the test microbial strains [19]. The supernatant-inoculated MHA plates were kept at 4 °C for at least 2 h to allow the diffusion of the antimicrobial metabolites. The diameters of inhibition zone were observed after 24 h incubation at 37 °C for bacteria and 28 °C for *C. albicans*. All the experiments were performed in triplicates.

### Disc diffusion assay

Twenty-four actinobacterial isolates that showed ≥ 25 mm diameter of inhibition zone against the MTCC strains in the agar well diffusion assay were further evaluated using disc diffusion assay [23]. To check more potential bioactive nature of the selected isolates, MRSA strain ATCC 43300 and the two clinical isolates CL1 and CL2 were also included in this assay. Crude culture extracts of the selected isolates were obtained by vigorously mixing the cell-free culture supernatant with ethyl acetate in 1:1 (*v*/v) ratio for 30 min, followed by separation of the organic layer in a separation funnel. The ethyl acetate extract was evaporated in a rotary evaporator (Rotavapor R-210, Buchi, Switzerland) under reduced pressure, and the crude extract was dissolved in 10% dimethyl sulphoxide (DMSO) to a final concentration of 1 mg/ml. Ten μl (10 μg) of each crude extract was loaded onto sterile filter paper discs (6 mm diameter) and air dried. The MHA plates, which were seeded separately with 10^8^ CFU of the test organisms, were loaded with the sterile discs impregnated with the crude extracts. The disc loaded with 10% DMSO was used as the negative control, while rifampicin (20 μg/disc) and amphotericin B (30 μg/disc) were used as the positive controls for bacteria and *C. albicans*, respectively. The diameters for the zone of inhibition were evaluated as performed above in the agar well diffusion assay. The mean value of inhibition diameter (arithmetic mean ± standard error of the mean) was calculated from the triplicates assay done for each isolate.

### Morphological characterization of actinobacterial strains

The selected 24 isolates were grown in Tyrosine Agar medium (ISP7) (M362, HiMedia) for 7 days to check their morphology like aerial and substrate mycelium. The spore colour was observed after 7–10 days of incubation.

### Genomic DNA extraction

The pure cultures of the actinobacterial isolates were grown in GLM medium at 28°C with shaking at 200 rpm for 3–4 days. The mycelia were then separated from the broth culture by centrifugation at 10000 rpm for 15 min at 4°C. One g of mycelium was aseptically crushed in mortar-pestle using liquid nitrogen. The crushed mycelium (~500 mg) was used for genomic DNA extraction using QIAamp DNA Mini Kit (Qiagen, Germany) following the manufacturer’s instructions. The DNA content was quantified spectrophotometrically using a nanodrop spectrophotometer. The extracted DNA was stored at − 20°C until required.

### Molecular identification of actinobacterial isolates

The 24 selected actinobacterial isolates were identified by 16S rRNA gene sequencing followed by a sequence similarity search. 16S rRNA gene was amplified using the universal eubacterial primers 27F (5′-AGAGTTTGATCCTGGCTCAG-3′) and 1492R (5′-GGTTACCTTGTTACGACTT-3′) [28]. Each 50 μl reaction volume contained 50 ng genomic DNA, 1X PCR reaction buffer, 1.5 mM MgCl_2_, 0.2 μM each primer, 0.2 mM each dNTP and 1 U Taq DNA polymerase (TaKaRa). The PCR reactions were performed in Proflex PCR System (Applied Biosystems, USA) using the following reaction conditions: initial denaturation at 94°C for 5 min, followed by 35 cycles at 94°C for 1 min 30 s, 54°C for 30 s and 72°C for 1 min, and final extension at 72°C for 10 min. The amplified fragments were analyzed by 1.5% (*w*/*v*) agarose gel electrophoresis and further purified using GenElute PCR Clean-Up Kit (Sigma Aldrich, USA) following manufacturer’s instructions. The purified amplicons were sequenced using the same amplification primers in an automated DNA sequencer by First BASE Laboratories, Malaysia. The raw sequences were analyzed to validate the base calls and the low quality base calls were trimmed out using Sequence Scanner 2.0 software (Applied Biosystems). The trimmed sequences were aligned manually to find overlap regions and contigs were generated. The assembled sequences were checked for the presence of chimera using DECIPHER v2 software [29]. To designate the taxonomic status of the isolates, the quality-checked, assembled sequences were queried against NCBI’s non-redundant, reference RNA sequence database (refseq_rna) in the nucleotide BLAST tool using the megablast algorithm. The isolates were assigned species-level taxonomy using the prescribed 98.7% similarity threshold [30].

### Detection of antimicrobial biosynthetic genes PKSII and NRPS

For the detection of antimicrobial biosynthetic genes and assessment of their genetic variations among the selected 24 isolates, the amplification of polyketide genes encoding β-ketosynthase domain of PKSII and adenylation domain of NRPS were performed using degenerate primer pairs KS-α (5′-TSGCSTGCTTCGAYGCSATC-3′)/KS-β (5′-TGGAANCCGCCGAABCCGCT-3′) [31] and A3F (5′-GCSTACSYSATSTACACSTCSGG-3′)/A7R (5′-SASGTCVCCSGTSCGGTAS-3′) [32] respectively. Each PCR reaction was carried out in a 10 μl reaction volume containing 25 ng genomic DNA, 1X PCR reaction buffer, 1.5 mM MgCl_2_, 0.2 μM each primer, 0.2 mM each dNTP and 1 U Taq DNA polymerase. Amplifications were carried out using the following reaction conditions: initial denaturation at 94°C for 7 min, followed by 35 cycles of 94°C for 1 min, 65°C (for PKSII) or 63°C (for NRPS) for 30 s and 72°C for 1 min, and a final extension at 72°C for 10 min. The amplified products were analyzed in 1.5% (*w*/*v*) low melting agarose gel. The desired bands of 600 bp for PKSII and 700 bp for NRPS genes were excised and purified. The purified amplicons were sequenced using the same amplification primers, and the sequences were analyzed and quality-checked as performed above. The nucleotide sequences were translated into amino acid sequences using the ORF Finder in NCBI [33]. The functional ORF sequences were queried against NCBI’s protein database using the BLASTP algorithm with default parameters.

### Phylogenetic evaluation

The identified 16S rRNA gene sequences and the CDS of the antimicrobial biosynthetic genes were aligned using Clustal X algorithm implemented in MEGA6 software along with the sequences of the nearest known taxa [34]. Neighbour joining tree was constructed based on the evolutionary distance calculated using Kimura-2-parameter substitution model. The consistency of the tree was verified by bootstrap scrutiny with 1000 resamplings using p-distance model.

### Minimum inhibitory concentration of EA-PWS52 against test microbial strains

Minimum inhibitory concentration (MIC) assay was performed as previously described [35, 36] with little modifications. A stock solution (3 mg/ml) of filter-sterilized (0.2 μm) ethyl acetate extract (EA-PWS52) of 7 days old culture of the isolate PWS52 was prepared in 10% dimethyl sulfoxide (DMSO). Different concentrations of the extract (10 μg - 80 μg) were transferred to a 96-well plate and 10^5^ CFU of freshly grown test microbial strains were added to each well. Ampicillin and fluconazole (5 μg each) were used as the positive controls, and 10% DMSO served as the negative control. The plates were aseptically incubated for 24 h at 37 °C for bacteria and 28 °C for fungus, and absorbance was measured at 620 nm using a UV-Vis spectrophotometer (Varioskan Flash, Thermo Scientific, San Jose, CA, USA). The plates were then incubated for 2 h at room temperature after adding 30 μl of 0.015% resazurin. The concentration at which no colour change was observed (blue) was considered as MIC value of the extract. Cells (10 μl) from the wells where blue colour was observed were spread on MHA and SDA plates and incubated for 24 h. The concentrations at which no visible bacterial or fungal growth was observed were noted as minimum bactericidal concentration (MBC) and minimum fungicidal concentration (MFC), respectively.

### Morphological effect of EA-PWS52 on the test microbial strains

Freshly grown MRSA (ATCC 43300) and *C. albicans* (MTCC 227) were respectively suspended on NB and SDB media to a final concentration of 10^6^ CFU/ml and were treated with EA-PWS52 at MIC for 16 h at optimum conditions. The cells were harvested by centrifugation at 4°C for 5 min and washed (two times) with phosphate buffer saline (PBS) (pH 7.4) solution. The cell pellets were fixed in 3% (*v*/v) glutaraldehyde in PBS and dehydrated in increasing concentrations of acetone (10%, v/v, increments, to 100%). Lastly, the cell pellets were critically dried with tetramethylsaline solution. Dehydrated cells were mounted onto stubs and coated with a film of gold for 15–20 min. The cells were viewed under a scanning electron microscope (SEM) (Carl Zeiss ∑igma VP) to obtain secondary electron images at an accelerating voltage of 15 kV and a probe diameter of 102 Pa.

### GC-MS analysis of EA-PWS52

Gas chromatography-mass spectrometry (GC-MS) analysis of EA-PWS52 was carried out in Shimadzu GC 2010 plus with triple quadrupole MS (TP-8030) machine as previously described [37]. The machine column used was EB-5MS of 30 m length, 0.25 μm thickness, and 25 mm internal diameter. Helium gas (1 ml/min) was used as the carrier gas to inject 0.2 μm-filtered samples at 270 °C. Temperature programme of the column was 40 °C for 5 min, followed by 10 °C/min increase in temperature to 200 °C. At this temperature, the column was kept isothermally for 10 min before increasing to 290 °C at 10 °C/min and kept for 5 min. Electron ionization mode was used to operate the mass spectrometer at 70 eV with a continuous scan from 45 to 600 m/z. Each constituent identification was performed by comparing the obtained mass spectra with the data available in the NIST library, USA. The structures of the detected metabolites were drawn using ChemDraw Ultra software.

### UHPLC analysis of EA-PWS52

The chromatographic separation of EA-PWS52 was carried out using a UHPLC-MS/MS 3100 UHPLC system (Thermo Scientific) following the rapid method previously described [38] with the following modifications. Five μl of the extract was injected into the column. Extract separation was carried out using a mobile phase of solvent A (water with 0.05% *v*/v formic acid) and solvent B (100% acetonitrile) at 25 °C with a constant flow rate of 0.3 ml/min. The full screen mass spectra from m/z 100–1500 amu was acquired with a resolution of 70,000. Orbitrap-MS was used for establishing exact mass measurements. Characterization of PWS52 extract constituents was done by comparing the observed m/z value of the sample with the literature and mass databases. Nalidixic acid was used as the standard.

### Accession numbers of nucleotide sequences

The 16S rRNA gene and functional ORF sequences obtained in the present study were submitted to the NCBI GenBank under the accession numbers: KU940237−KU940250, KX171763−KX171770, KX255002 and KX255003 for 16S rRNA gene, and KX592592−KX592594, KX575651, KX761862 and KX575648 for ORF sequences of PKSII and NRPS genes.

## Results

### Isolation of actinobacteria

To assess the bioactive actinobacteria (specially *Streptomyces* sp*.*) in the two ecosystems, selective isolation was carried out from 14 composite soil samples representing diverse habitats using ISP2, SA, AIA and GLM media supplemented with rifampicin, nalidixic acid and amphotericin B. A total of 172 morphologically distinct presumptive actinobacterial isolates were obtained from different ecological niches of NNP and PWS such as naturally grown tree and grass rhizospheric soil, forest litter and swampy area (Table 1). Maximum number of isolates were recovered from SA medium (*n* = 67) followed by GLM (*n* = 45), AIA (*n* = 32) and ISP2 (*n* = 28).

**Table 1:** Actinobacterial isolates obtained from Nameri National Park (NNP) and Panidehing Wildlife Sanctuary (PWS), Assam, India and their antimicrobial activities

Sample collection sites	No. of isolates in different isolation media				Total no. of isolates	No. of isolates showing antimicrobial activity against test pathogens (%)^a,b^			Isolates with antimicrobial activity against SA, EC and CA (%)^c^	Isolates with antimicrobial activity (≥ 25 mm IZ) against SA, EC and CA (%)^c,d^
	AIA	ISP2	GLM	SA		SA	EC	CA		
NNP	17	19	26	44	106	58 (54.72)	52 (49.06)	26 (24.53)	21 (19.81)	14 (13.21)
PWS	15	9	19	23	66	28 (42.42)	22 (33.33)	12 (18.18)	12 (18.18)	10 (15.15)
Total	32	28	45	67	172	86 (50.00)	74 (43.02)	38 (22.09)	33 (19.19)	24 (13.95)

^a^SA: *S*. *aureus* MTCC 96; EC: *E*. *coli* MTCC 40; CA: *C. albicans* MTCC 227

^b^Screening of antimicrobial activity was done by spot inoculation method. The % of active isolates were calculated based on the total number of isolates obtained in each collection sites

^c^Screening of antimicrobial activity was done by agar well diffusion method. The % of active isolates were calculated based on the total number of isolates obtained in each collection sites

^d^IZ: Inhibition zone

### In vitro screening of antimicrobial activity

**Fig. 1:**
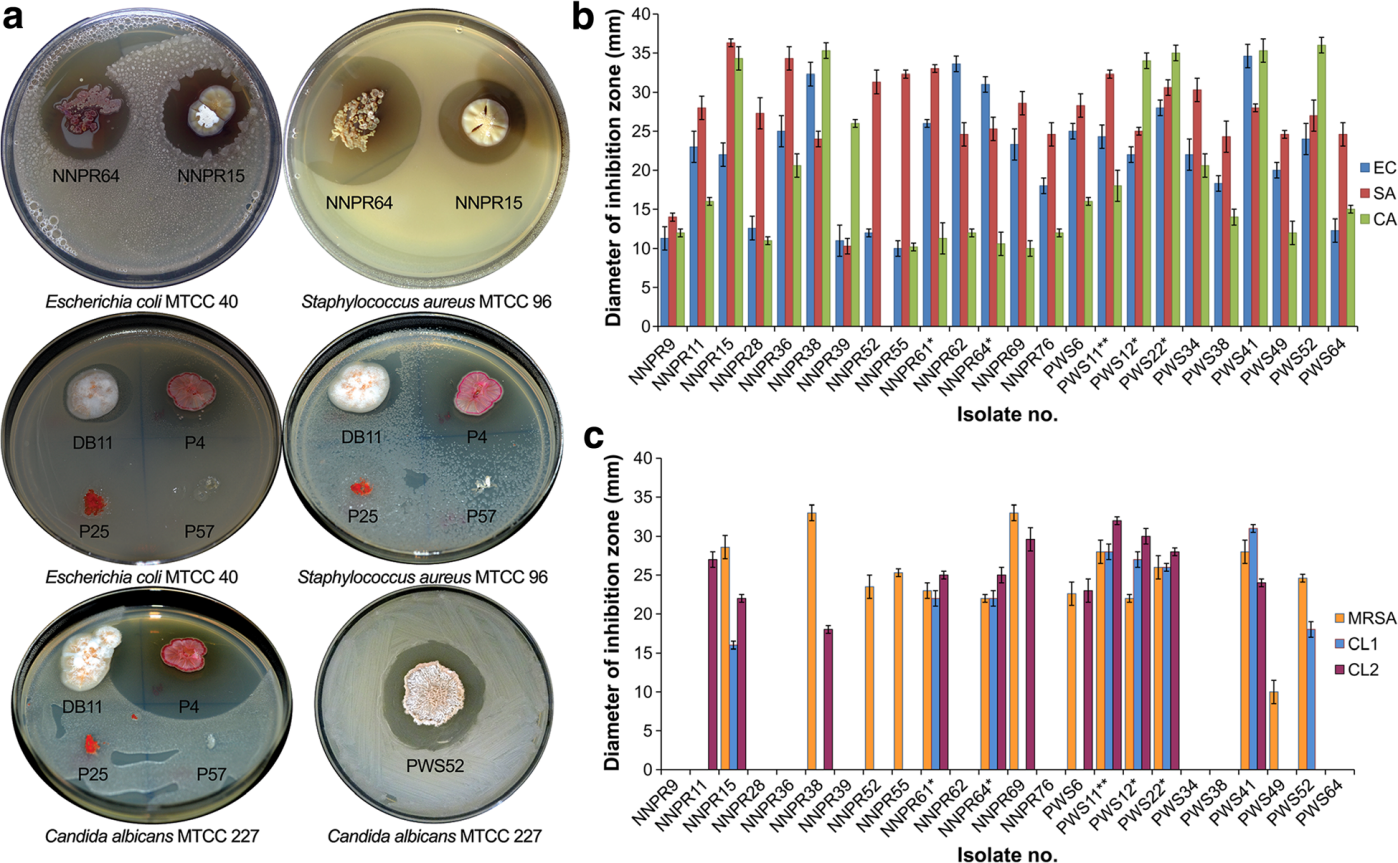
**a** Screening of antimicrobial activity of actinobacteria; **b** the antimicrobial activities of the selected 24 actinobacterial isolates against MTCC test pathogens and **c** antimicrobial activity against MRSA and antibiotic-resistant clinical isolates. **a** The figure shows representative antimicrobial actinobacterial isolates. Screening was done by spot inoculation. **b**, **c** Antimicrobial activities were determined by disc diffusion assay. The graph represents the activity of ethyl acetate extract of cell-free culture supernatant. The diameter of inhibition zone was determined after loading pathogen-seeded MHA plates with ethyl acetate extract impregnated (10 μg) filter paper discs followed by 24 h incubation at 37°C for bacteria and 28°C for *C. albicans*. Each bar represents arithmetic mean of three independent replicates and the error bar indicates the standard error of the mean. In x-axis, single asterisk (*) after the isolate number represents the detected presence of PKSII antimicrobial biosynthetic gene, while double asterisks (**) indicates the detected presence of both PKSII and NRPS antimicrobial biosynthetic genes. EC: *Escherichia coli* MTCC 40, SA: *Staphylococcus aureus* MTCC 96, CA: *Candida albicans* MTCC 227, MRSA: Methicillin-resistant *Staphylococcus aureus* ATCC 43300, CL1: Clinical isolate *Staphylococcus saprophyticus* CL1, Clinical isolate *Bacillus pumillus* CL2

All the putative actinobacterial isolates were preliminarily screened for their ability to produce antimicrobial metabolites against the Gram-positive *S*. *aureus*, Gram-negative *E*. *coli* and yeast *C. albicans* using the spot inoculation technique (Fig. 1a). The antimicrobial metabolites producing actinobacterial isolates were predominantly recovered from NNP than PWS (Table 1). Among the 172 isolates, 96 isolates (55.81%) exhibited antimicrobial activity against at least one of the test microbial strains, 86 isolates (50%) produced antimicrobial substances against *S*. *aureus* MTCC 96, 74 isolates (43.02%) were active against *E*. *coli* MTCC 40 and 38 isolates (22.09%) showed antagonistic activity against *C*. *albicans* MTCC 227 (Table 1).

To confirm the production of extracellular antimicrobial metabolites, the 96 bioactive isolates were subjected to submerged fermentation and the crude cell-free culture supernatants were used for agar well diffusion assay against the test microbial strains. Though most of the tested isolates that showed antimicrobial activity in spot inoculation assay also showed similar activities, some of the isolates showed no antimicrobial activity. The isolates that showed antimicrobial activity against the three test microbial strains were selected for further evaluation and analysis. Thirty-three isolates (19.19%) exhibited both antibacterial and antifungal activities (Table 1). Interestingly, an unexpectedly high number of isolates that showed antagonistic activity against *C*. *albicans* (MTCC 227) (NNP: 21 out of 26 isolates, 80.77%; PWS: all the 12 isolates, 100%) showed antibacterial activity. In contrast, comparatively less number of isolates with antibacterial activity against *S*. *aureus* MTCC 96 (NNP: 21 out of 58 isolates, 36.21%; PWS: 12 out of 28 isolates, 42.86%) and *E*. *coli* MTCC 40 (NNP: 21 out of 52 isolates, 40.38%; PWS: 12 out of 22 isolates, 54.55%) had also antifungal activity. Based on the higher degree (zone of inhibition) of both antifungal and antibacterial metabolites production, 24 isolates out of these 33 isolates exhibited efficient antimicrobial activity with a diameter of inhibition zone ranging from 25 to 45 mm. These 24 isolates were further evaluated for their bioactivity using the ethyl acetate extracts of the culture supernatants by disc diffusion assay. All the selected 24 isolates showed antimicrobial activity against *S. aureus* MTCC 96, *E. coli* MTCC 40 and *C*. *albicans* MTCC 227 except isolate NNPR52, which showed no antifungal activity (Fig. 1b). Overall, the highest activity against the three test microbial strains (> 25 mm) was shown by isolates PWS22 (28.0 ± 1.0 mm to 35.0 ± 1.0 mm) and PWS41 (28.0 ± 1.5 mm to 35.3 ± 1.5 mm). Among the isolates originated from NNP, maximum antimicrobial activity was recorded for NNPR15, NNPR36 and NNPR38 (20.6 ± 1.5 mm to 36.3 ± 0.5 mm). In the similar activity range, five isolates from PWS (PWS12, PWS22, PWS34, PWS41 and PWS52) showed potential antimicrobial activity. The highest inhibitions of *S. aureus* MTCC 96, *E. coli* MTCC 40 and *C. albicans* MTCC 227 were achieved by PWS41 (34.6 ± 1.5 mm), NNPR15 (36.3 ± 0.5 mm) and PWS52 (36.0 ± 1.0 mm), respectively. The antimicrobial efficacies of the ethyl acetate extracts were also tested against antibiotic-resistant strains viz. MRSA (ATCC 43300) and two clinical isolates *S. saprophyticus* (CL1) and *B. pumilus* (CL2). Interestingly, these antibiotic-resistant strains were resistant to the antimicrobial metabolites produced by 9 isolates. The remaining 15 isolates showed antimicrobial activity in at least one of the antibiotic-resistant strains and they were more active towards MRSA (14 isolates) followed by *B. pumilus* CL2 (11 isolates) and *S. saprophyticus* CL1 (8 isolates) (Fig. 1c). These active isolates inhibiting the antibiotic-resistant strains mostly originated from PWS (7 out of 10 isolates representing 70% recovery) as compared to NNP (8 out of 14 isolates representing 57.14% recovery). This indicated that the various unexplored soil habitats of PWS might harbor actinobacteria that could be the potential sources for discovery of novel antimicrobial compounds against antibiotic-resistant pathogens. Isolates NNPR38 and NNPR69 showed maximum activity (33.0 ± 1.0 mm) against test strain MRSA, while the highest inhibition of *S. saprophyticus* CL1 and *B. pumilus* CL2 were recorded for PWS41 (31.0 ± 0.5 mm) and PWS11 (32.0 ± 0.5 mm), respectively. Out of the 15 isolates, 7 isolates showed activities against all six test strains, with the maximum broad range activity (> 25 mm) recorded for only 02 isolates, PWS11 (28.0 ± 1.5 mm to 32.0 ± 2.0 mm) and PWS22 (28.0 ± 1.0 mm to 28.0 ± 1.0 mm). These potential antimicrobial activities might be attributed to the diversity in the structure of the antimicrobial metabolites as well as the associated diverse mechanisms of action.

### Identification of bioactive actinobacteria

**Fig. 2:**
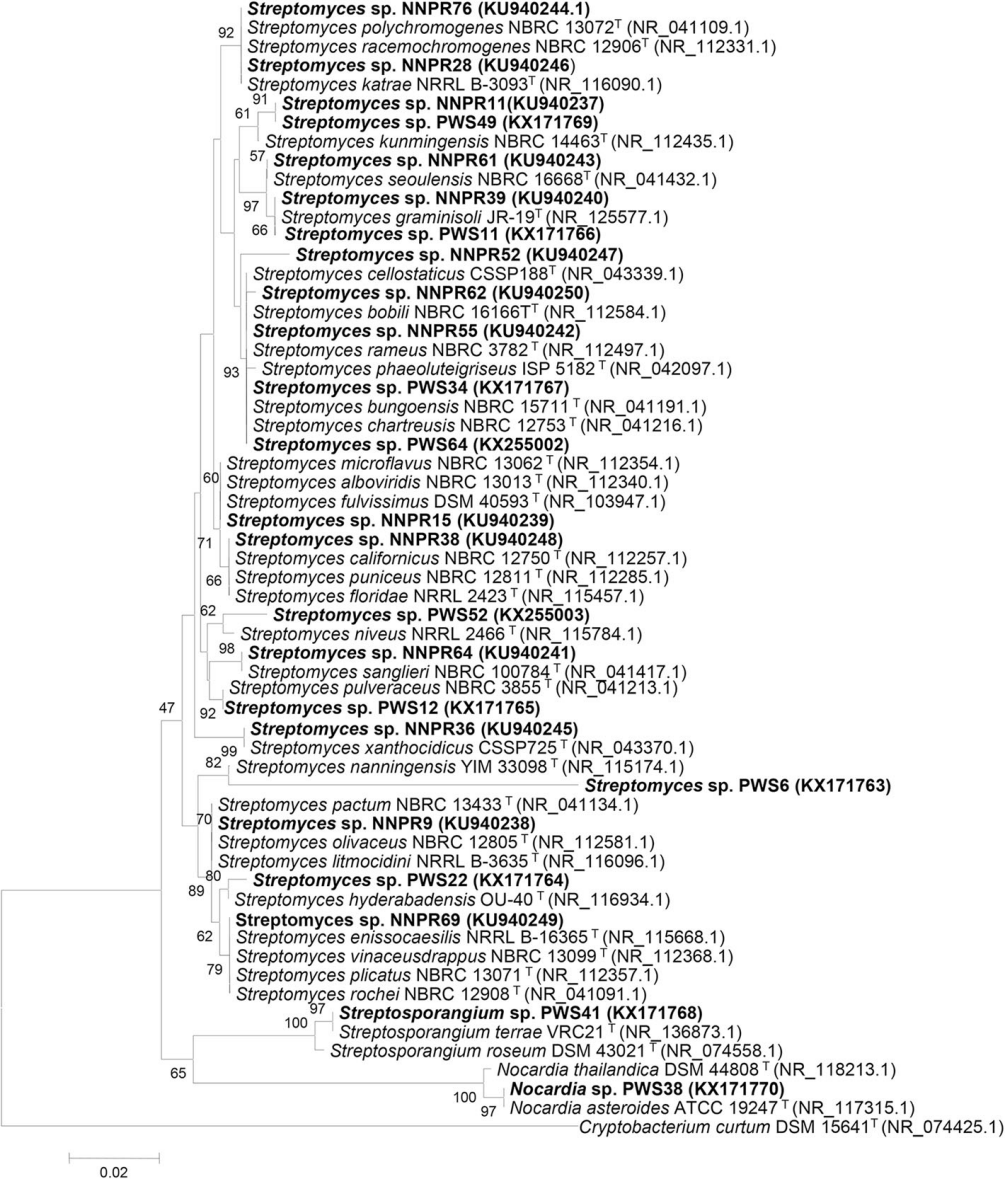
16S rRNA gene-based phylogenetic tree showing evolutionary relationships of the 24 actinobacterial isolates possessing antimicrobial activity with their closest known taxa. The evolutionary history was inferred using the Neighbor-Joining method. The tree was constructed based on the evolutionary distances calculated using Kimura 2-parameter substitution model. All positions containing gaps and missing data were eliminated. The percentage of replicate trees in which the associated taxa clustered together in the bootstrap test (1000 replicates) are shown next to the branches for values > 40%. The bar represents 0.02 substitutions per nucleotide position (2% sequence divergence). GenBank accession numbers are mentioned within the parentheses. *Cryptobacterium curtum* was the outgroup in the analysis. ^T^ = Type strain

The initial identification of the 24 presumptive antimicrobial actinobacterial isolates was done through colony morphology observation, which is typical of actinobacteria such as slow growing, aerobic, glabrous or chalky, folded and aerial and substrate mycelia of different colours (Additional file 1: Table S1, Additional file 2: Figure S1). In addition, the majority of pure cultures grown in agar slants possessed an earthy odour. The presumptive isolates were assigned taxonomic status by sequencing of partial or near full-length 16S rRNA gene, followed by BLAST search in the NCBI’s non-redundant, reference RNA sequence database of valid species. The isolates predominantly belonged to the genus *Streptomyces* (22 isolates, 91.67%), followed by *Nocardia* (01 isolate, 4.17%) and *Streptosporangium* (01 isolate, 4.17%) (Additional file 3: Table S2). Thirteen isolates were identified up to species level while the remaining 11 isolates up to genus level. Phylogenetic analysis of nearly full-length 16S rRNA gene sequences (> 1300 bp) revealed the presence of considerable nucleotide variations in three isolates from their closest related strains (PWS6: 40 substitutions, 1 insertion and 33 deletions; NNPR11: 9 substitutions, 3 insertions and 3 deletions; PWS49: 7 substitutions, 4 insertions and 3 deletions), and they formed distinct phylogenetic clades from their closest known taxa (Fig. 2). Apart from this, isolates NNPR9, NNPR15, NNPR62, NNPR69 and NNPR76 showed very high species-level similarity (> 99.5%) with more than one species at the same similarity score, threshold and E-value, thus creating ambiguity in the strain identification (Additional file 3: Table S2). This is also reflected in the phylogenetic tree where the isolates showed separate distinct clusters together with their closest known taxa (Fig. 2).

### Detection of antimicrobial biosynthetic genes

**Fig. 3:**
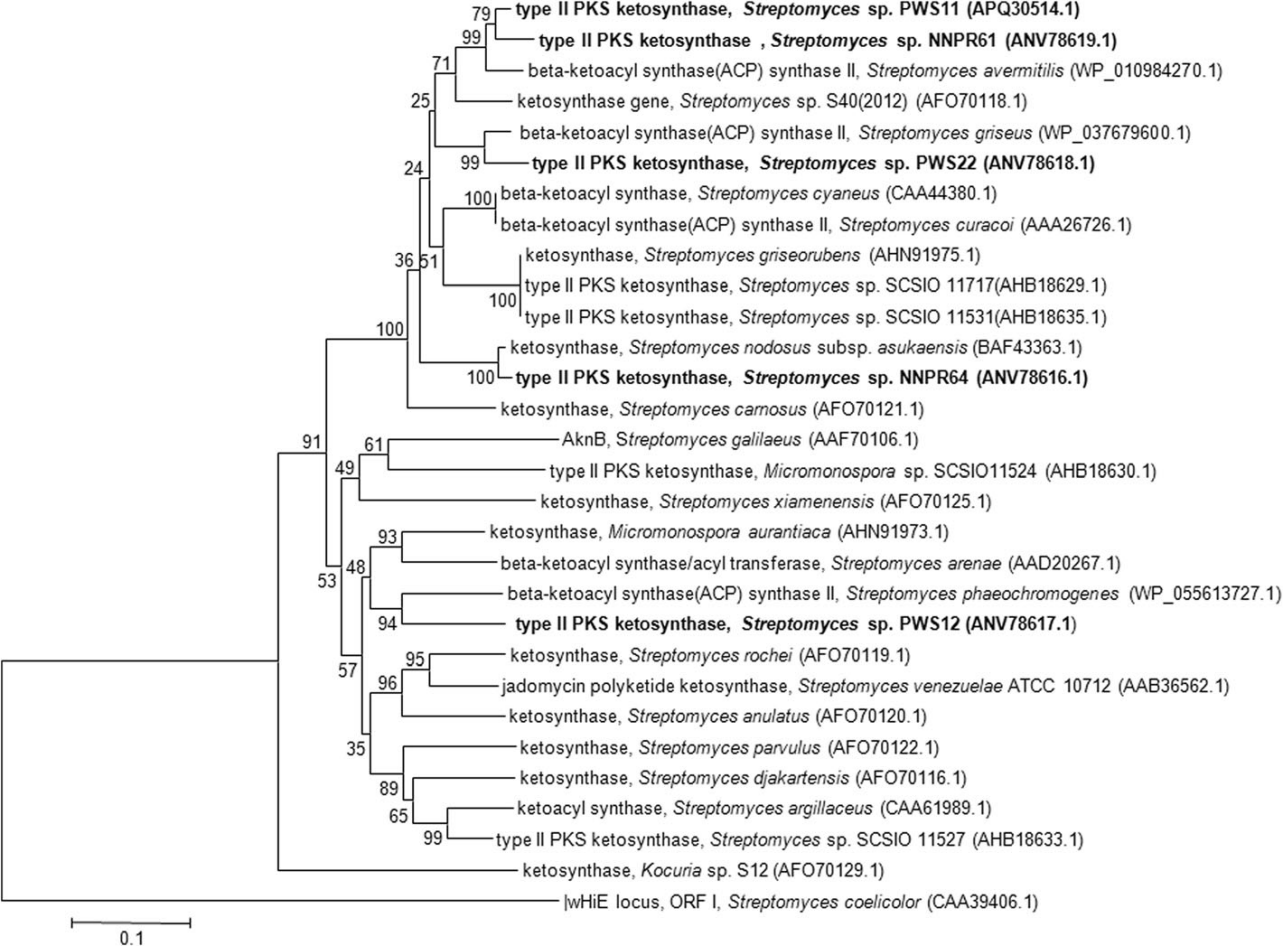
Dendrogram based on the partial coding sequence of the antimicrobial biosynthetic gene PKSII of the selected actinobacterial isolates. Values at nodes indicate bootstrap support (%) for the branch points at 1000 resampling. The bar represents 0.1 substitutions per nucleotide position. GenBank accession numbers are mentioned within the parentheses

The antimicrobial activities of the 24 isolates were correlated with the presence of antimicrobial biosynthetic genes. Only five bioactive isolates showed the presence of PKSII and NRPS genes (Fig. 1b, c). Interestingly, all these isolates showed antimicrobial activity against all the test microbial strains. The translated amino acid sequences of the PKSII genes showed 82–97% similarity with their closest known counterparts of the genus *Streptomyces* (Table 2, Fig. 3). The PKSII gene obtained from NNPR61, PWS11, PWS12 and PWS22 strains showed close similarity to beta-ACP synthase gene while the counterpart of NNPR64 was closely related with ketosynthase gene. Only isolate PWS11 showed the presence of NRPS gene.

**Table 2:** Antimicrobial biosynthetic genes detected in the antimicrobial actinobacterial isolates

Strain	Taxonomy	Gene	Top BLAST match	Query coverage (%)	Identity %	BLAST pathway product	NCBI GenBank accession no.
NNPR61	*Streptomyces seoulensis*	PKSII	Beta ACP synthase from *Streptomyces avermitis* (WP_010964270)	98.0	93.0	Spore pigment	KX592592, ANV78619.1
NNPR64	*Streptomyces sanglieri*	PKSII	Ketosynthase from *Streptomyces nodosus* subsp. *asukaensis* (BAF43363)	93.0	97.0	Manumycin-type metabolites	KX575651, ANV78616.1
PWS11	*Streptomyces graminisoli*	PKSII	Beta ACP synthase from *Streptomycesavermitis* (WP_010964270)	99.0	94.0	Spore pigment	KX761862, APQ30514.1
		NRPS	Amino acid adenylation protein from *Streptomyces antibioticus* (WP_059190418)	98.0	92.0	Polypeptide-type metabolites	KX575648
PWS12	*Streptomyces pulveraceus*	PKSII	Beta ACP synthase from *Streptomyces phaeochromogenes* (WP_055613727)	98.0	82.0	Benzoisochromanequinone-type metabolites	KX592594, ANV78617.1
PWS22	*Streptomyces* sp.	PKSII	Beta ACP synthase from *Streptomyces griseus* (WP_037679600)	99.0	94.0	Spore pigment	KX592593 ANV78618.1

### Minimum inhibitory concentration of EA-PWS52 against test microbial strains

In order to highlight the antimicrobial potential of the active actinobacterial isolates, analysis of antimicrobial metabolites and interaction with the test microbial strains were performed using the active fraction of a potent antimicrobial metabolite producing isolate. The key criterion for the selection of the isolate from amongst the members of potent antimicrobial metabolite producers was the lowest similarity in taxonomic identity with the closest known taxa based on 16S rRNA gene sequence. Isolate PWS52 showed the lowest 16S rRNA gene sequence similarity (97.7%) with the closest known taxa (Additional file 3: Table S2), which is far below the indicated threshold of 98.7% similarity for species level identification [30]. The taxonomic identity of PWS52 was also ambiguous as it matched with *Streptomyces niveus* NRRL 2466 (T) (GenBank accession no. NR115784.1) and *Streptomyces pulveraceus* NBRC 3855 (T) (GenBank accession no. NR_041213.1) at the same maximum score, query coverage, E-value and similarity of the top BLAST hits. Hence, the ethyl acetate extract of PWS52 (EA-PWS52), which showed antimicrobial activity against *S. aureus*, *E.coli*, MRSA, clinical strain CL1, and *C. albicans,* was selected for the detailed analysis of its bioactivity. To study the interaction of EA-PWS52 with the test microbial strains, MIC of the extract was evaluated. The MIC for *E. coli* was 20 μg, followed by *S. aureus* (30 μg) and MRSA (40 μg). *C. albicans* and strain CL1 had the same MIC value at 50 μg. The representative image of 96 well plate is shown in Additional file 4: Figure S2.

### Morphological effect of EA-PWS52 on the test microbial strains

**Fig. 4:**
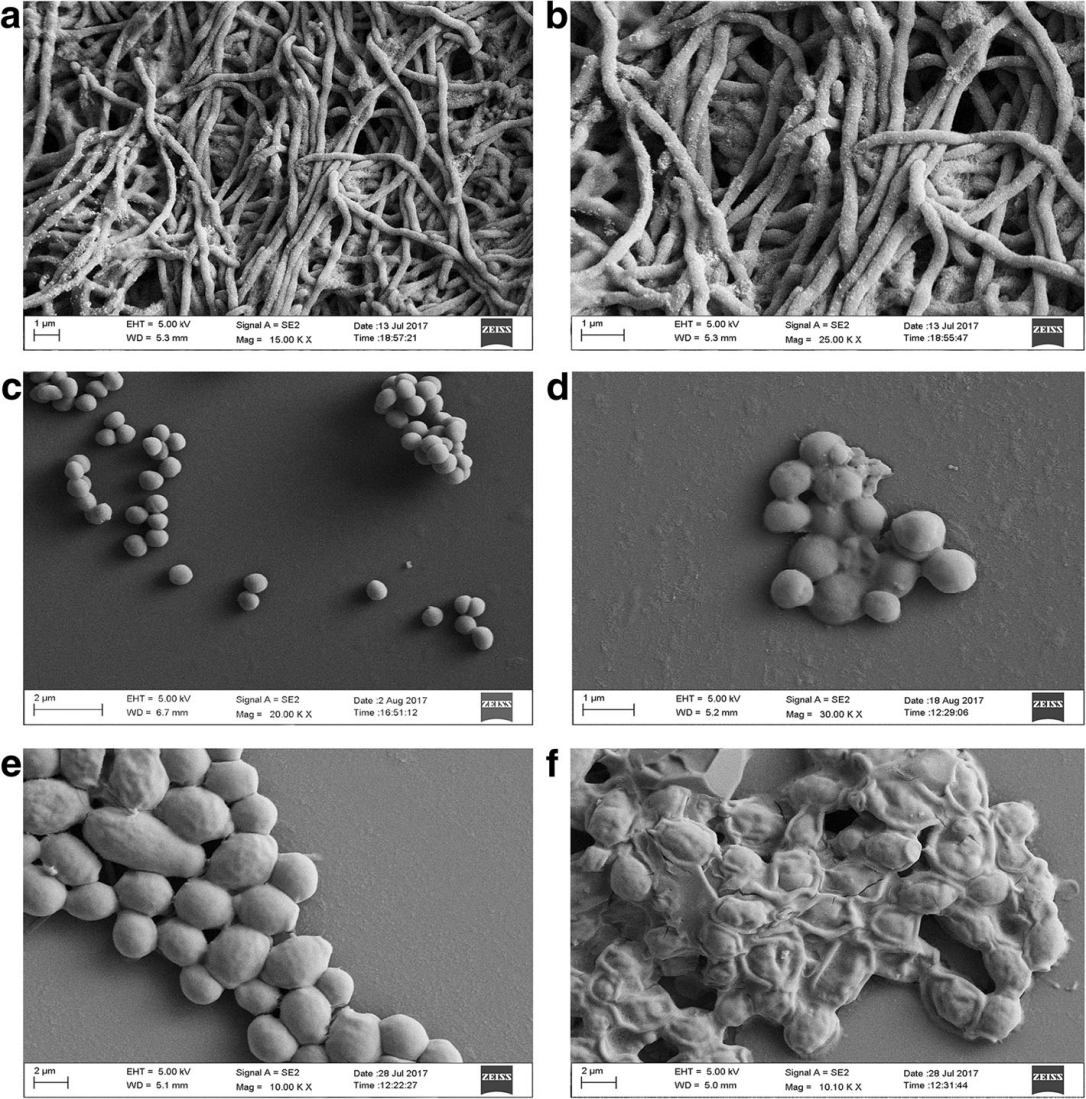
Interaction of antimicrobial extract (EA-PWS52) of *Streptomyces* sp. PWS52 with test strains. **a**, **b** SEM images of *Streptomyces* sp. PWS52 at different magnifications. **c** SEM image of control, untreated cells of MRSA. **d** SEM image of MRSA after treatment with EA-PWS52, clearly showing cell swelling and changes in morphology. **e** Control, untreated cells of *C. albicans* under SEM. **f** SEM images of cells of *C. albicans* after treatment with EA-PWS52, clearly showing cell deformation and shrinkage

SEM analysis of MRSA and *C. albicans* cells after treatment with EA-PWS52 showed a significant difference in the morphology in comparison with the control cells (Fig. 4). Treated cells of MRSA showed very little growth in culture broth and the treatment deformed the bacterial cells. In case of yeast, control cells were intact and had maintained their actual cell morphology, whereas treated cells lost their cell integrity due to shrinkage.

### GC-MS analysis of EA-PWS52

GC-MS analysis revealed 2,5-bis(1,1-dimethylethyl) phenol and benzeneacetic acid as the major metabolites present in the EA-PWS52 covering a peak area of 21.11 and 10.22%, respectively (Table 3). Other nine compounds viz. 1-Nonadecene, Dibutyl-phthalate, 7,9-Di-tert-butyl-1-oxaspiro(4,5)deca-6,9-diene-2,8-dione, 1-Heptacosanol, Phthalic acid, hex-3-yl isobutyl ester, Isopropyl myristate, Heptacosyl heptafluorobutyrate, Pthalic acid, di(2-propylpentyl) ester and Pyrrolo[1,2-a]pyrazine-1,4-dione,hexahydro-3-(2-methylpropyl) were also detected in different retention time. The EA-PWS52 in total acquired seven constituents, which are known to produce antifungal, antibacterial and antioxidant activities. This precisely verified the bioactive activities that were shown during the antimicrobial screening. The structures of the metabolites obtained from the GC-MS analysis are given in Additional file 5: Figure S3.

**Table 3:** Compounds identified using GC-MS from EA-PWS52 of *Streptomyces* sp. PWS52

Sl. no.	Retention time (min)	Compound	Molecular weight (MW)	Area %	Reported activity	References
1	20.098	2,5-bis(1,1-dimethylethyl) Phenol	206	21.11	Antifungal, Antioxidant, Antimicrobial	[64, 65, 70]
2	16.433	Benzeneacetic acid	136	10.22	Antifungal, Antimicrobial	[66, 67]
3	24.217	1-Nonadecene	266	8.95	Antifungal, Antimicrobial	[69]
4	28.123	Dibutyl phthalate	278	6.61	Antimicrobial	[71]
5	26.933	7,9-Di-tert-butyl-1-oxaspiro(4,5)deca-6,9-diene-2,8-dione	276	3.95	No reported antimicrobial	This study
6	35.18	1-Heptacosanol	396	3.39	Antimicrobial, Antioxidant	[72]
7	25.631	Phthalic acid, hex-3-yl isobutyl ester	320	2.72	No reported antimicrobial	This study
8	24.725	Isopropyl myristate	270	2.37	Antimicrobial, Antioxidant	[73]
9	40.539	Heptacosyl heptafluorobutyrate	592	1.51	No reported antimicrobial	This study
10	39.928	Phthalic acid, di(2-propylpentyl) ester	390	1.49	No reported antimicrobial	This study
11	27.352	Pyrrolo[1,2-a]pyrazine-1,4-dione,hexahydro-3-(2-methylpropyl)	210	1.42	Antimicrobial	[68]

### UHPLC analysis of EA-PWS52

**Fig. 5:**
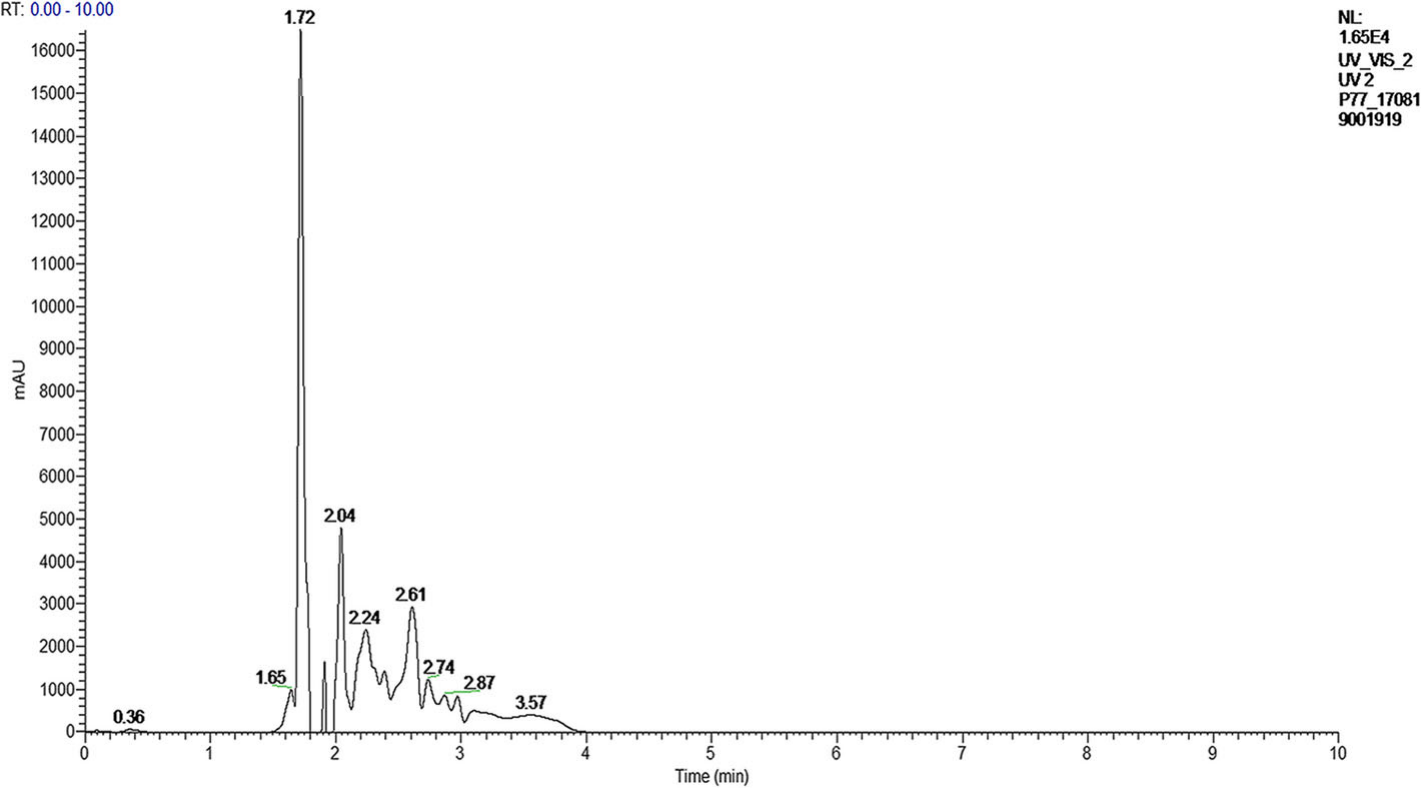
UHPLC chromatogram of EA-PWS52 at 270 nm

Chromatographic analysis using UHPLC-PDA was done to detect the antimicrobial compounds present in EA-PWS52. The chromatogram of the EA-PWS52 is shown in Fig. 5. The peaks present in EA-PWS52 chromatogram were identified by comparing the retention time (RT; min), λ max and mass spectra with the standard compounds reported in the literature using LC-ESI-MS/MS. The standard nalidixic acid was detected at λ max 270 nm, RT: 1.75 min (negative mode), [M-H]^¯^ ion at m/z 231.08 and compared with the EA-PWS52 chromatogram. The peaks with a retention time of 1.72 min and 2.04 min were identified as nalidixic acid and flumequine, respectively. The molecular masses of the constituent compounds present in EA-PWS52 were matched with previously described antimicrobial compounds (Additional file 6: Figure S4).

## Discussion

Actinobacteria are filamentous Gram-positive bacteria with high G + C content in their genomes. A large percent of their genome is known for the production of bioactive metabolites [39]. Besides that, there has been a decrease in the search for antimicrobial metabolites in recent years. Therefore, the probability of finding new and efficient bioactive metabolites from actinobacteria can be increased by continuing the search in underexplored areas [40]. Northeast India harbors diverse forest ecosystems that are still poorly explored and require scientific interventions to search for biologically active microorganisms for the production of antimicrobial metabolites though few researches have been conducted [19, 23–26]. No scientific reports are available from NNP and PWS soil systems about the antimicrobial metabolite producing actinobacteria. This is the first study where actinobacterial strains isolated from NNP and PWS were explored for the production of antimicrobial metabolites against bacterial and fungal pathogens.

Among actinobacteria, *Streptomyces* species are the major producers of antimicrobial metabolites. The isolation media were selected based on our previous experience and literature reports [11, 23]. Addition of antibacterial and antifungal agents had been confirmed to be a good strategy to eliminate the occurrence of contaminating microorganisms, thereby facilitating the growth of slow growing actinobacteria. A total of 172 phenotypical different actinomycetes were isolated, where more phenotypically diverse actinobacterial isolates were recovered from NNP soil samples in comparison to the PWS samples. This may be due to the collection of samples from the wetlands that are seasonally flooded with water. Although a small number of samples were used for the isolation of actinobacteria from NNP and PWS, many phenotypically diverse presumptive actinobacterial isolates were successfully isolated, indicating the existence of rich microbial diversity within the forest ecosystems. Forest litter soil samples collected from NNP and PWS showed maximum phenotypical diversity in comparison with other sample collection sites such as grassland and swampy areas (data not shown).

Out of the 172 actinobacterial isolates 96 isolates showed antimicrobial activity against at least one of the test microbial strains. This finding is similar to the previous antimicrobial studies, which showed considerably high rate of recovery of actinobacteria with antibacterial and antifungal activities [23, 41, 42]. Our result indicated that the soil habitats of NNP and PWS forest ecosystems appeared to be a rich source of antimicrobial metabolites producing actinobacteria, highlighting the importance of screening their microbial diversity. In spot inoculation assay, more number of actinomycetes isolates showed antibacterial activity against *S. aureus*. Out of 96 isolates, 33 isolates were able to produce both antibacterial and antifungal secondary metabolites during submerged fermentation. More number of actinobacterial isolates that had antifungal activity also exhibited antibacterial activity, but few isolates had only antibacterial activity. These findings confirmed that the actinobacteria could produce an array of a variety of antimicrobial metabolites that might be specifically antibacterial or broad spectrum in nature. These forest ecosystems are protected from human activity that may have contributed to the production of previously unexplored and powerful metabolites.

The identification based on 16S rRNA gene revealed that *Streptomyces* spp. were dominantly isolated from the soil samples of NNP and PWS. This finding is congruent with earlier studies, which reported that the percentage of recovery of *Streptomyces* spp. with antimicrobial activity was higher than other actinobacterial genera [41]. As observed during morphological diversity, NNP and PWS harbored distinctly different actinobacterial diversity with very little (only 2 isolates out of 24 isolates) sharing of culturable actinobacterial species (NNPR11/PWS49; NNPR39/PWS11). Four isolates (NNPR52, NNPR62, PWS6 and PWS52) had less than 98% similarity to its nearest known strain, which suggested that these isolates could be novel taxa [30] This is supported by the description of various phylogenetically similar (> 99%) *Streptomyces* strains as new and novel species [43–45]. Intragenomic heterogeneity, intraspecific disparity, inadequate resolution capacity and inconsistent DNA–DNA relatedness limit species-level delineation by 16S rRNA gene sequence data within the genus *Streptomyces* [46–48]. In fact, a definitive 16S rRNA gene sequence similarity threshold cannot be defined to describe whether an actinobacterium belonging to *Streptomyces* may or may not be a novel species [49]. Thus, the promising antimicrobial metabolites producing actinobacteria obtained in the present study demand a thorough investigation using polyphasic molecular and chemotaxonomic approach to ascertain the novel species status.

*Streptomyces* spp. are known for the production of polyketides and nonribosomal polyketide compounds, which have significant use in the medicinal community [31]. In the present study, five isolates gave positive amplification of the beta-ACP synthase and ketosynthase genes of PKSII gene cluster and one isolate showed positive amplification of the adenylation domain of NRPS gene (Table 2, Fig. 3). These genes and domains are known for the production of pigments and bioactive secondary metabolites in *Streptomyces.* The ketosynthase gene of *Streptomyces luteosporeus* S40, which is the closest related protein sequence homolog of PKSII gene of the isolate NNPR61, was predicted to produce spore pigment [50]. The translated protein sequence of PKSII gene from NNPR64 showed 97% similarity with the ketosynthase gene of *S. nodusus* subsp*. aukaensis* that is known to produce the antimicrobial and antitumor agent asukamycin, a member of manumycin family metabolites [51]. The PKSII gene partial CDS from PWS12 was most closely related to the beta-ACP synthase gene of *S. phaeochromogenes*, which is reported to produce benzoisochromanequinones type of having antimicrobial properties [52]. Isolate PWS11 also showed the presence of NRPS gene, its closest similarity being the adenylation domain of NRPS gene in *S. antibioticus* that is associated with the production of a polypeptide class of antibiotic, actinomycin [53, 54]. Discovering a new drug or an antimicrobial secondary metabolite is a time consuming process and it also demands a good amount of economic support. This can be reduced by bioprospecting the presence and diversity of PKS and NRPS biosynthetic gene clusters among different bioactive *Streptomyces* spp. from different habitats.

Apart from contributing to the production of antimicrobial secondary metabolites, PKS genes also play a key role in making pathogenic actinobacteria resistant to host defence mechanisms. *Mycobacterium tuberculosis* and related mycobacteria species possess highly lipid-rich cell envelope containing a repertoire of lipidic polyketides, known as mycoketides, that provides resistance to host mechanisms of recognition and killing by functioning as an immunoprotective barrier [55]. Analysis of *Mycobacterium* genome revealed that PKS12 encoded the largest mycoketide in *M. tuberculosis,* and it contributed to the cell wall’s drug transporting abilities that functioned in multidrug-resistant *Mycobacterium avium* [56, 57]. In *Mycobacterium marinum*, two novel type III PKSs, MMAR_2470 and MMAR_2474, synthesized methylated polyketides that were vital for the survival of the pathogen through biofilms, thereby providing a secure niche for developing multidrug resistance [55]. The PKS1 of *M. tuberculosis* contributed to the formation of infection-causing biofilms that predisposed to modulate the host immune system [58].

Though PKS genes possess contrasting functions in different actinobacteria, the antimicrobial metabolites of actinobacterial origin have antagonistic activities against mycobacterial pathogens. A novel thiopeptide compound, nocardithiocin, isolated from *Nocardia pseudobrasiliensis* strain IFM 0757 showed potent antimycobacterial activity against an array of antibiotic-resistant *Mycobacterium* strains [59]. Manikkam et al. reported diverse isolates of *Streptomyces* and non-*Streptomyces* spp. from less explored terrestrial ecosystems that were highly active against *M. tuberculosis* [60]. Previously we had reported isolation and characterization of a broad spectrum antibiotic, 2-methylheptyl isonicotinate, from *Streptomyce*s sp. 201 obtained from tea garden soil in Jorhat, Assam, India and it exhibited promising bioactivity against a wide range of fungal pathogens and *M. tuberculosis* [61, 62]. In our screening programme of new bioactive metabolites from actinobacteria, we had reported the broad spectrum antimicrobial activity of *Nocardia* sp. PB-52 isolated from the soil of Pobitora Wildlife Sanctuary of Assam, India [19].

In our study, the antimicrobial extract EA-PWS52 was found to contain phenolic compounds and benzeneacetic acid as dominant metabolites. Phenolic compounds are known for their antimicrobial and antioxidant activities [63]. The 2,5-bis(1,1-dimethylethyl)phenol compound was found in the highest percentage amount in the EA-PWS52 and was shown to have efficient antifungal and antibacterial activities [64, 65]. Benzeneacetic acid showed antifungal activity against phyto pathogens [66], whereas it also had antimicrobial activity against infectious human pathogens like *S. aureus* and *E.coli* [67]. The EA-PWS52 also consisted of pyrrolizidines [pyrrolo[1,2-a]pyrazine-1,4-dione,hexahydro-3-(2-methylpropyl)], which are natural heterocyclic compounds known to exhibit antimicrobial activity [68]. Another compound present in the EA-PWS52 in good amount was 1-nonadecene, which was characterized as a bioactive compound having antifungal and antibacterial activities [69]. The antimicrobial activity shown by the isolate PWS52 against the test microbial strains might be due to the synergistic effect of the metabolites present in the EA-PWS52. UHPLC-PDA report of EA-PWS52 detected nalidixic acid as the major antimicrobial compounds, similar to the earlier reports [9, 35]. Presence of all these influential antimicrobial compounds made the *Streptomyces* strain PWS52 a potent antimicrobial metabolite producer that can be further studied for other bioactivities against different disease-causing microorganisms.

## Conclusion

The present study showed that the microbiologically unexplored soil habitats of the NNP and PWS ecosystems harbor potential bioactive actinobacteria, especially *Streptomyces*, capable to produce an array of bioactive extracellular secondary metabolites that are specifically antibacterial or antifungal towards antibiotic-non-resistant and antibiotic-resistant pathogens. Over 55% of the actinobacterial isolates obtained exhibited antimicrobial activity against at least one of the tested pathogens. Although 24 isolates showed production of antimicrobial metabolites with efficient antimicrobial activity, only few isolates could be considered as effective antimicrobial metabolites producers based on their potential bioactivity towards both test microbial strains and antibiotic-resistant test strains. Further, strain PWS52 might be a novel strain with potent antimicrobial activity, which can be explored for more antimicrobial metabolites against various other pathogens in the future. The potential antimicrobial actinobacteria isolated from both NNP and PWS soil ecosystems can be further studied to characterize and identify the lead bioactive compounds from the respective isolates, and confirm their effectiveness in both in vitro and in vivo models while contributing to the global effort to fight against rising antibiotic-resistance.

## Additional files

Additional file 1: **Table S1.** “Morphological characteristics of some of the actinobacterial isolates obtained in the present study.” (DOCX 14 kb)

Additional file 2: **Figure S1.** “Colony morphological diversity of some of the 24 presumptive antimicrobial actinobacterial isolates.” (PDF 1711 kb)

Additional file 3: **Table S2.** “Molecular identification of the 24 selected antimicrobial actinobacterial isolates based on 16S rRNA gene sequencing.” (DOCX 20 kb)

Additional file 4: **Figure S2.** “96 well plate showing minimum inhibitory concentration (MIC) of EA-PWS52 extract against MRSA test microbial strain.” In this figure, wells present in rows B, C, D (column 1) are negative control and contain only MRSA cells. Rows B, C, D (column 2) are also negative control having MRSA cells and 10% DMSO, which also showed presence of viable cells (change of resazurin reagent colour from blue to pink). Column 3 to 10 (rows B, C, D) contain different concentrations of EA-PWS52 extract among which column 5 (purple in colour) shows decrease in cell viability. As column 6 has no change in colour (blue) of resazurin reagent, the corresponding concentration was taken as the MIC of EA-PWS52 extract against MRSA. Rows F, G, H (column 1) are positive controls, contain MRSA cells and ampicillin, and show no viable cells as the reagent colour (blue) has not changed. (DOCX 4199 kb)

Additional file 5: **Figure S3.** “Chemical structures of metabolites present in PWS52 extract analyzed through GC-MS.” (PDF 1535 kb)

Additional file 6: **Figure S4.** “Mass spectra of (A) nalidixic acid (B) flumequine present in PWS52 extract, (C) standard nalidixic acid (m/z : 233.09) in positive mode and (D) standard nalidixic acid (m/z: 231.08) in negative mode.” (PDF 1499 kb)

## Abbreviations

AGC: Automatic gain control
AIA: Actinomycete Isolation Agar
DMSO: Dimethyl sulphoxide
EA-PWS52: Ethyl acetate extract of isolate PWS52
GC-MS: Gas Chromatography-Mass Spectrometry
ISP2: International *Streptomyces* Project Yeast Malt Agar medium
MBC: Minimum bactericidal concentration
MFC: Minimum fungicidal concentration
MHA: Mueller Hinton Agar
MHB: Muller Hilton Broth
MIC: Minimum inhibitory concentration
MRSA: Methicillin-resistant *Staphylococcus aureus*
NA: Nutrient Agar
NB: Nutrient Broth
NNP: Nameri National Park
NRPS: Nonribosomal peptide synthetase
OD: Optical density
PBS: Phosphate buffer saline
PKSII: Polyketide synthase type II
PWS: Panidehing Wildlife Sanctuary
SA: Streptomyces Agar
SDA: Sabouraud Dextrose Agar
SDB: Sabouraud Dextrose Broth
SEM: Scanning electron microscope
