# Supplementary material for: Antimicrobial potentiality of actinobacteria isolated from two microbiologically unexplored forest ecosystems of Northeast India
Source: BMC Microbiol. 2018 Jul 11;18:71. doi: 10.1186/s12866-018-1215-7 (PMC6042205; doi:10.1186/s12866-018-1215-7)
Supplement: Supplementary file 1 — Table S1. “Morphological characteristics of some of the actinobacterial isolates obtained in the present study.” (DOCX 14 kb) [file 12866_2018_1215_MOESM1_ESM.docx]

**Additional file 1: Table S1** Morphological characteristics of some of the actinobacterial isolates obtained in the present study

| **Isolates** | **Taxonomy^a^** | **Aerial mycelia** | **Substrate mycelia** | **Spore colour** | **Soluble pigmentation** | **Media** | **Habitat** |
| --- | --- | --- | --- | --- | --- | --- | --- |
| NNPR9 | *Streptomyces* sp. | white | dark brown | grey | nil | AIA | NNP |
| NNPR11 | *Streptomyces kunmingensis* | peach | cream | peach | nil | AIA | NNP |
| NNPR15 | *Streptomyces* sp. | white | cream | white | nil | SA | NNP |
| NNPR28 | *Streptomyces* sp. | dark brown | black | white | nil | ISP2 | NNP |
| NNPR36 | *Streptomyces*  *xanthocidicus* | white | black | grey | brown | GLM | NNP |
| NNPR38 | *Streptomyces* sp. | white | white | brown | nil | SA | NNP |
| NNPR39 | *Streptomyces graminisoli* | cream | brown | white | brown | SA | NNP |
| NNPR52 | *Streptomyces* sp. | cream | cream | white | nil | AIA | NNP |
| NNPR55 | *Streptomyces rameus* | dark brown | dark brown | nil | brown | SA | NNP |
| NNPR61 | *Streptomyces seoulensis* | white | cream | nil | nil | ISP2 | NNP |
| NNPR62 | *Streptomyces* sp. | brown | black | grey | nil | GLM | NNP |
| NNPR64 | *Streptomyces sanglieri* | dark brown | dark brown | nil | dark brown | SA | NNP |
| NNPR69 | *Streptomyces* sp. | white | white | white | nil | ISP2 | NNP |
| NNPR76 | *Streptomyces* sp. | brown | dark brown | white | nil | SA | NNP |
| PWS6 | *Streptomyces* sp. | brown | brown | white | brown | GLM | PWS |
| PWS11 | *Streptomyces graminisoli* | grey | cream | nil | nil | SA | PWS |
| PWS12 | *Streptomyces pulveraceus* | brown | black | brown | nil | SA | PWS |
| PWS22 | *Streptomyces* sp. | white | white | nil | nil | SA | PWS |
| PWS34 | *Streptomyces chartreusis* | grey | brown | white | nil | AIA | PWS |
| PWS38 | *Nocardia asteroids* | cream | cream | grey | nil | SA | PWS |
| PWS41 | *Streptosporangium terrae* | yellow | cream | nil | nil | GLM | PWS |
| PWS49 | *Streptomyces kunmingensis* | cream | cream | white | light brown | AIA | PWS |
| PWS52 | *Streptomyces* sp. | brown | brown | white | nil | AIA | PWS |
| PWS64 | *Streptomyces cellostaticus* | cream | cream | white | orange | SA | PWS |

^a^Taxonomic identification of the isolates was done by 16S rRNA gene sequencing. See Additional file 3: Table S2 for more details.
