# Supplementary material for: Antimicrobial potentiality of actinobacteria isolated from two microbiologically unexplored forest ecosystems of Northeast India
Source: BMC Microbiol. 2018 Jul 11;18:71. doi: 10.1186/s12866-018-1215-7 (PMC6042205; doi:10.1186/s12866-018-1215-7)
Supplement: Supplementary file 2 — Figure S1. “Colony morphological diversity of some of the 24 presumptive antimicrobial actinobacterial isolates.” (PDF 1711 kb) [file 12866_2018_1215_MOESM2_ESM.pdf]

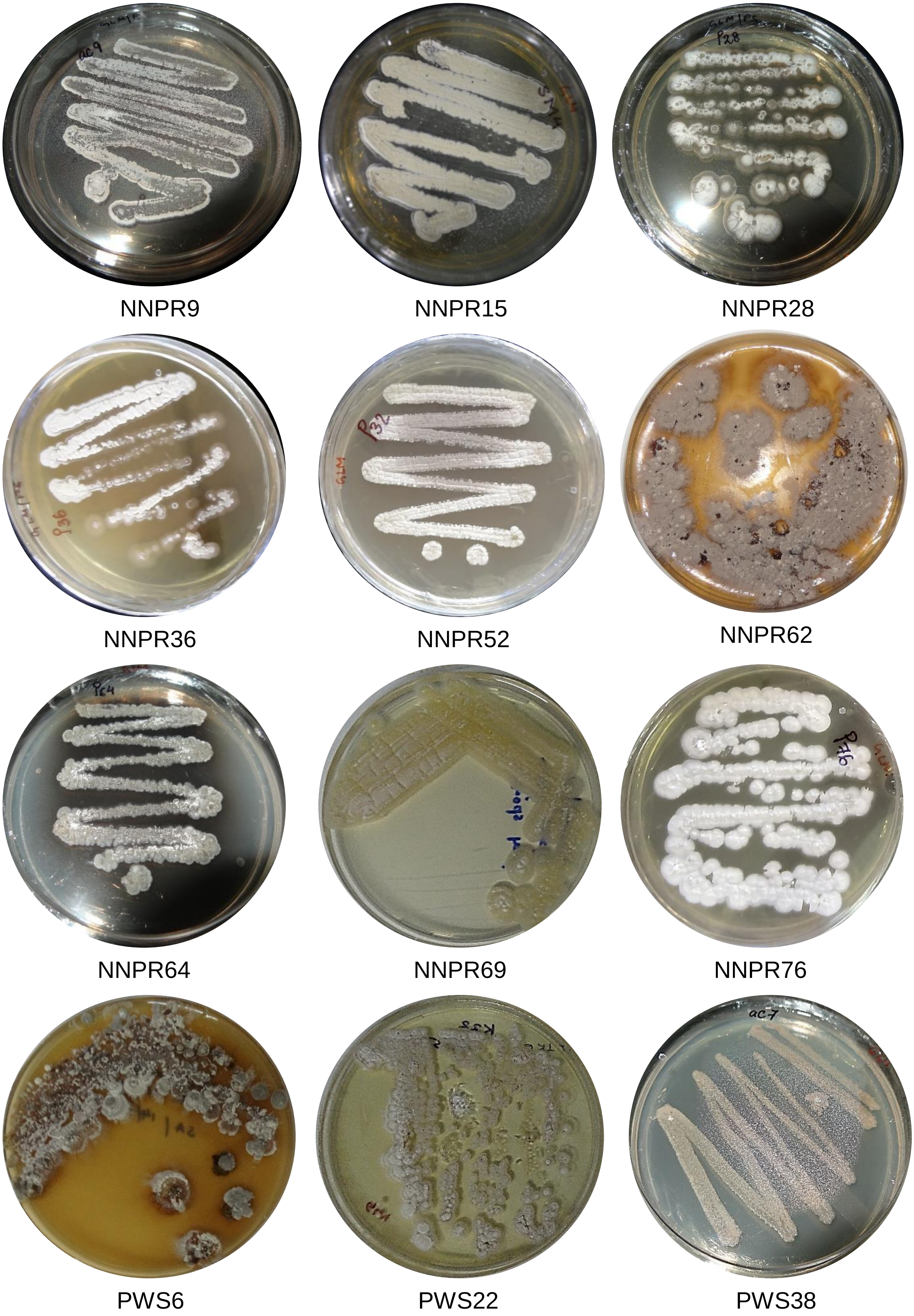

Additional file 2: Figure S1 Colony morphological diversity of some of the 24 presumptive antimicrobial actinobacterial isolates.
