# Supplementary material for: Antimicrobial potentiality of actinobacteria isolated from two microbiologically unexplored forest ecosystems of Northeast India
Source: BMC Microbiol. 2018 Jul 11;18:71. doi: 10.1186/s12866-018-1215-7 (PMC6042205; doi:10.1186/s12866-018-1215-7)
Supplement: Supplementary file 3 — Table S2. “Molecular identification of the 24 selected antimicrobial actinobacterial isolates based on 16S rRNA gene sequencing.” (DOCX 20 kb) [file 12866_2018_1215_MOESM3_ESM.docx]

**Additional file 3: Table S2** Molecular identification of the 24 selected antimicrobial actinobacterial isolates based on 16S rRNA gene sequencing.

| **Isolate name** | **Analyzed sequence length (bp)** | **Closest known species^a^** | **Max Score** | **Query Coverage (%)** | **E -value** | **Similarity (%)** | **Identification^b^** | **NCBI GenBank accession number** |
| --- | --- | --- | --- | --- | --- | --- | --- | --- |
| NNPR9 | 1228 | *Streptomyces litmocidini* NRRL B-3635 (T) (NR_116096.1) / *Streptomyces olivaceus* NBRC 12805 (T) (NR_112581.1) / *Streptomyces pactum* NBRC 13433 (T) (NR_041134.1) | 2242 | 100 | 0.0 | 99.7 | *Streptomyces* sp. | KU940238 |
| NNPR11 | 1312 | *Streptomyces kunmingensis* NBRC 14463 (T) (NR_112435.1) | 2342 | 100 | 0.0 | 98.9 | *Streptomyces kunmingensis* | KU940237 |
| NNPR15 | 1193 | *Streptomyces fulvissimus* DSM 40593 (T) (NR_103947.1) / *Streptomyces microflavus* NRRL B-2156 (T) (NR_043854.1) / *Streptomyces alboviridis* JCM 4449 (T) (NR_115374.1) | 2198 | 100 | 0.0 | 99.9 | *Streptomyces* sp. | KU940239 |
| NNPR28 | 697 | *Streptomyces katrae* NRRL B-3093 (T) (NR_116090.1) / *Streptomyces polychromogenes* NBRC 13072 (T) (NR_041109.1) / *Streptomyces racemochromogenes* NBRC 12906 (T) (NR_112331.1) | 1280 | 99 | 0.0 | 99.9 | *Streptomyces* sp. | KU940246 |
| NNPR36 | 1335 | *Streptomyces xanthocidicus* CSSP725 (T) (NR_043370.1) | 2218 | 92 | 0.0 | 99.1 | *Streptomyces xanthocidicus* | KU940245 |
| NNPR38 | 760 | *Streptomyces californicus* NBRC 12750 (T) (NR_112257.1) / *Streptomyces puniceus* NBRC 12811 (T) (NR_112285.1) / *Streptomyces floridae* NRRL 2423 (T) (NR_115457.1) | 1399 | 100 | 0.0 | 99.9 | *Streptomyces* sp. | KU940248 |
| NNPR39 | 1246 | *Streptomyces graminisoli* JR-19 (T) (NR_125577.1) | 2290 | 100 | 0.0 | 99.8 | *Streptomyces graminisoli* | KU940240 |
| NNPR52 | 726 | *Streptomyces bungoensis* NBRC 15711 (T) (NR_041191.1) | 1230 | 99 | 0.0 | 97.5 | *Streptomyces* sp. | KU940247 |
| NNPR55 | 1038 | *Streptomyces rameus* NBRC 3782 (T) (NR_112497.1) | 1886 | 100 | 0.0 | 99.5 | *Streptomyces rameus* | KU940242 |
| NNPR61 | 1218 | *Streptomyces seoulensis* NBRC 16668 (T) (NR_041432.1) | 2231 | 100 | 0.0 | 99.8 | *Streptomyces seoulensis* | KU940243 |
| NNPR62 | 1147 | *Streptomyces bobili* NBRC 16166T (T) (NR_112584.1) / *Streptomyces phaeoluteigriseus* ISP 5182 (T) (NR_042097.1) | 1873 | 94 | 0.0 | 97.7 | *Streptomyces* sp. | KU940250 |
| NNPR64 | 1349 | *Streptomyces sanglieri* NBRC 100784 (T) (NR_041417.1) | 2464 | 99 | 0.0 | 99.7 | *Streptomyces sanglieri* | KU940241 |
| NNPR69 | 1239 | *Streptomyces rochei* NRRL B-1559 (T) (NR_116078.1) / *Streptomyces enissocaesilis* NRRL B-16365 (T) (NR_115668.1) / *Streptomyces vinaceusdrappus* NBRC 13099 (T) (NR_112368.1) / *Streptomyces plicatus* NBRC 13071 (T) (NR_112357.1) | 2080 | 91 | 0.0 | 99.6 | *Streptomyces* sp. | KU940249 |
| NNPR76 | 1286 | *Streptomyces polychromogenes* NBRC 13072 (T) (NR_041109.1) / *Streptomyces racemochromogenes* NBRC 12906 (T) (NR_112331.1) | 2185 | 92 | 0.0 | 99.7 | *Streptomyces* sp. | KU940244 |
| PWS6 | 1367 | *Streptomyces nanningensis* YIM 33098 (T) (NR_115174.1) | 2121 | 98 | 0.0 | 94.7 | *Streptomyces* sp. | KX171763 |
| PWS11 | 1370 | *Streptomyces graminisoli* JR-19 (T) (NR_125577.1) | 2525 | 100 | 0.0 | 99.9 | *Streptomyces graminisoli* | KX171766 |
| PWS12 | 1372 | *Streptomyces pulveraceus* NBRC 3855 (T) (NR_041213.1) | 2518 | 100 | 0.0 | 99.8 | *Streptomyces pulveraceus* | KX171765 |
| PWS22 | 714 | *Streptomyces hyderabadensis* OU-40 (T) (NR_116934.1) | 1243 | 99 | 0.0 | 98.0 | *Streptomyces* sp. | KX171764 |
| PWS34 | 1312 | *Streptomyces chartreusis* NBRC 12753 (T) (NR_041216.1) | 2418 | 100 | 0.0 | 99.9 | *Streptomyces chartreusis* | KX171767 |
| PWS38 | 1210 | *Nocardia asteroides* ATCC 19247 (T) (NR_117315.1) | 2235 | 100 | 0.0 | 100.0 | *Nocardia asteroids* | KX171770 |
| PWS41 | 1365 | *Streptosporangium terrae* VRC21 (T) (NR_136873.1) | 2433 | 100 | 0.0 | 98.8 | *Streptosporangium terrae* | KX171768 |
| PWS49 | 1338 | *Streptomyces kunmingensis* NBRC 14463 (T) (NR_112435.1) | 2394 | 100 | 0.0 | 99.0 | *Streptomyces kunmingensis* | KX171769 |
| PWS52 | 1107 | *Streptomyces niveus* NRRL 2466 (T) (NR115784.1) / *Streptomyces pulveraceus* NBRC 3855 (T) (NR_041213.1) | 1875 | 98 | 0.0 | 97.7 | *Streptomyces* sp. | KX255003 |
| PWS64 | 1224 | *Streptomyces cellostaticus* CSSP188 (T) (NR043339.1) | 2209 | 100 | 0.0 | 99.3 | *Streptomyces cellostaticus* | KX255002 |

^a^ Closest hit was determined using NCBI’s nucleotide BLAST tool against the non-redundant, reference RNA sequence database (refseq_rna). Species name is followed by the strain number, type strain (T) and GenBank accession number.

^b^ Isolates with similarity value greater than 98.7% [30] and with only one closest hit at the same identity parameters are classified up to species level.
