# Supplementary material for: Antimicrobial potentiality of actinobacteria isolated from two microbiologically unexplored forest ecosystems of Northeast India
Source: BMC Microbiol. 2018 Jul 11;18:71. doi: 10.1186/s12866-018-1215-7 (PMC6042205; doi:10.1186/s12866-018-1215-7)
Supplement: Supplementary file 4 — Figure S2. “96 well plate showing minimum inhibitory concentration (MIC) of EA-PWS52 extract against MRSA test microbial strain.” In this figure, wells present in rows B, C, D (column 1) are negative control and contain only MRSA cells. Rows B, C, D (column 2) are also negative control having MRSA cells and 10% DMSO, which also showed presence of viable cells (change of resazurin reagent colour from blue to pink). Column 3 to 10 (rows B, C, D) contain different concentrations of EA-PWS52 extract among which column 5 (purple in colour) shows decrease in cell viability. As column 6 has no change in colour (blue) of resazurin reagent, the corresponding concentration was taken as the MIC of EA-PWS52 extract against MRSA. Rows F, G, H (column 1) are positive controls, contain MRSA cells and ampicillin, and show no viable cells as the reagent colour (blue) has not changed. (DOCX 4199 kb) [file 12866_2018_1215_MOESM4_ESM.docx]

**Additional file 4: Figure S2** 96 well plate showing minimum inhibitory concentration (MIC) of EA-PWS52 extract against MRSA test microbial strain. In this figure, wells present in rows B, C, D (column 1) are negative control and contain only MRSA cells. Rows B, C, D (column 2) are also negative control having MRSA cells and 10% DMSO, which also showed presence of viable cells (change of resazurin reagent colour from blue to pink). Column 3 to 10 (rows B, C, D) contain different concentrations of EA-PWS52 extract among which column 5 (purple in colour) shows decrease in cell viability. As column 6 has no change in colour (blue) of resazurin reagent, the corresponding concentration was taken as the MIC of EA-PWS52 extract against MRSA. Rows F, G, H (column 1) are positive controls, contain MRSA cells and ampicillin, and show no viable cells as the reagent colour (blue) has not changed.
