# Supplementary material for: Antimicrobial potentiality of actinobacteria isolated from two microbiologically unexplored forest ecosystems of Northeast India
Source: BMC Microbiol. 2018 Jul 11;18:71. doi: 10.1186/s12866-018-1215-7 (PMC6042205; doi:10.1186/s12866-018-1215-7)
Supplement: Supplementary file 5 — Figure S3. “Chemical structures of metabolites present in PWS52 extract analyzed through GC-MS.” (PDF 1535 kb) [file 12866_2018_1215_MOESM5_ESM.pdf]

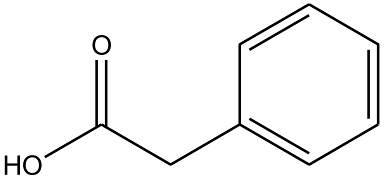

Benzeneacetic acid

(1)

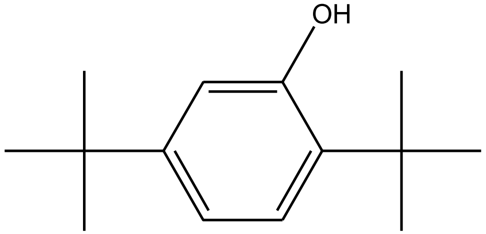

2,5-bis(1,1-dimethylethyl) Phenol

(2)

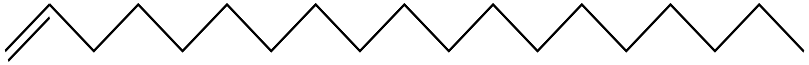

1-Nonadecene

(3)

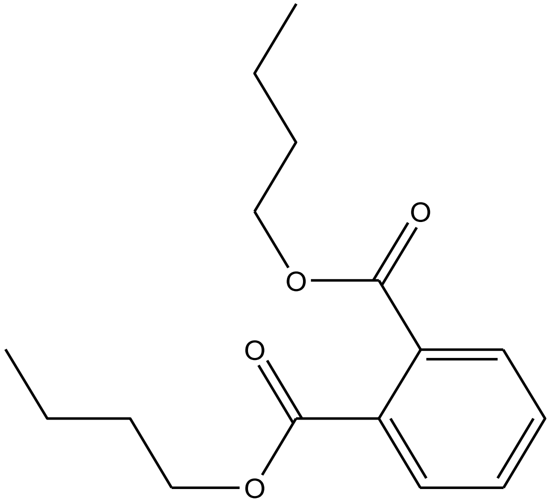

Dibutyl phthalate

(4)

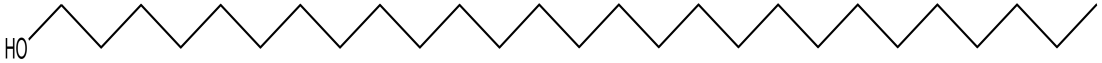

1- Heptacosanol

(5)

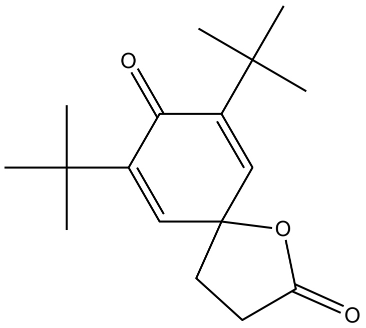

7,9-Di-tert-butyl-1-oxaspiro(4,5)deca-6,9-diene-2,8-dione

(6)

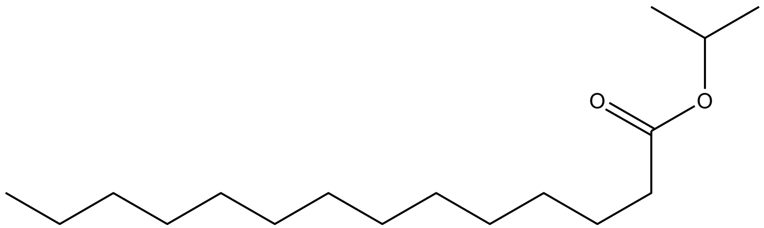

Isopropyl myristate

(7)

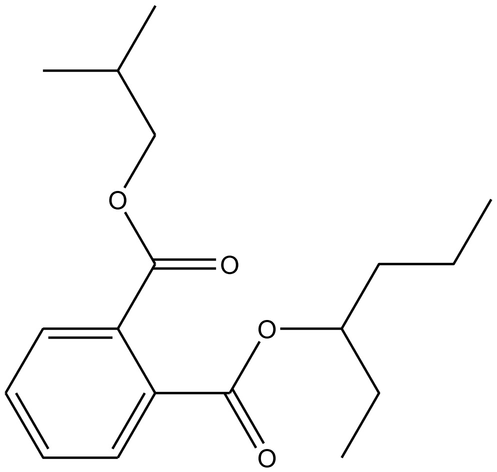

Phthalic acid, hex-3-yl isobutyl ester

(8)

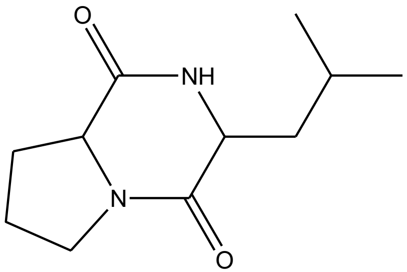

Pyrrolo[1,2-a]pyrazine-1,4-dione,
hexahydro-3-(2-methylpropyl)

(9)

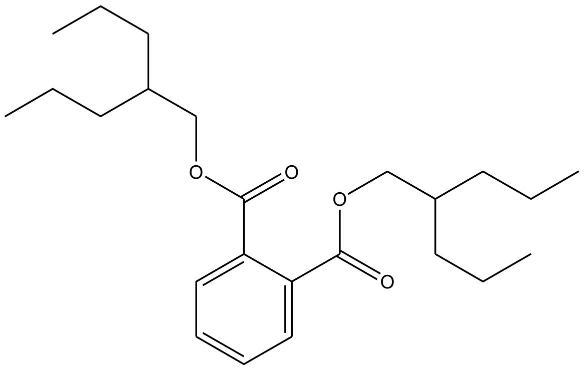

Phthalic acid, di(2-propylpentyl) ester

(10)

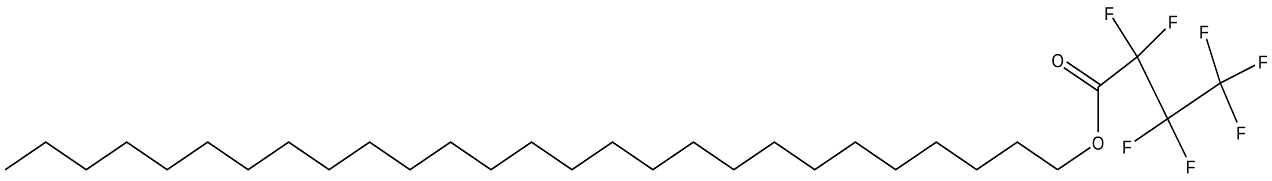

Heptacosyl heptafluorobutyrate

(11)

Additional file 5: Figure S3 Chemical structures of metabolites present in PWS52 extract analysed through GC-MS.
