# Supplementary material for: Antimicrobial potentiality of actinobacteria isolated from two microbiologically unexplored forest ecosystems of Northeast India
Source: BMC Microbiol. 2018 Jul 11;18:71. doi: 10.1186/s12866-018-1215-7 (PMC6042205; doi:10.1186/s12866-018-1215-7)
Supplement: Supplementary file 6 — Figure S4. “Mass spectra of (A) nalidixic acid (B) flumequine present in PWS52 extract, (C) standard nalidixic acid (m/z : 233.09) in positive mode and (D) standard nalidixic acid (m/z: 231.08) in negative mode.” (PDF 1499 kb) [file 12866_2018_1215_MOESM6_ESM.pdf]

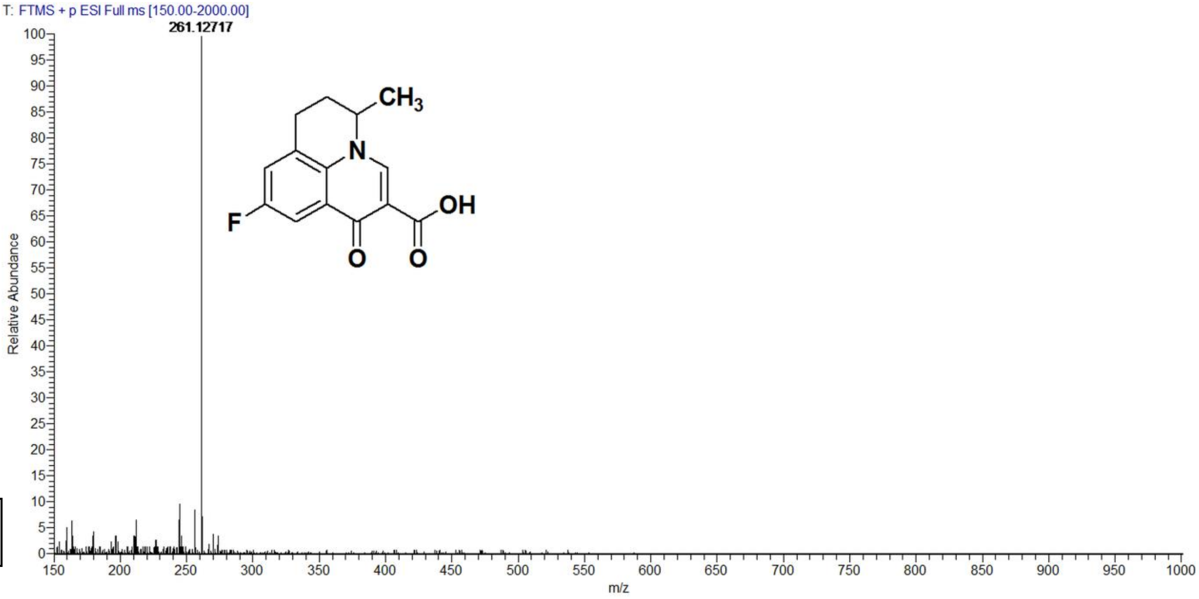
T: FTMS + p ESI Full ms [150.00-2000.00]
261.12717
CH3
N
F
OH
O
O
Relative Abundance
m/z

A

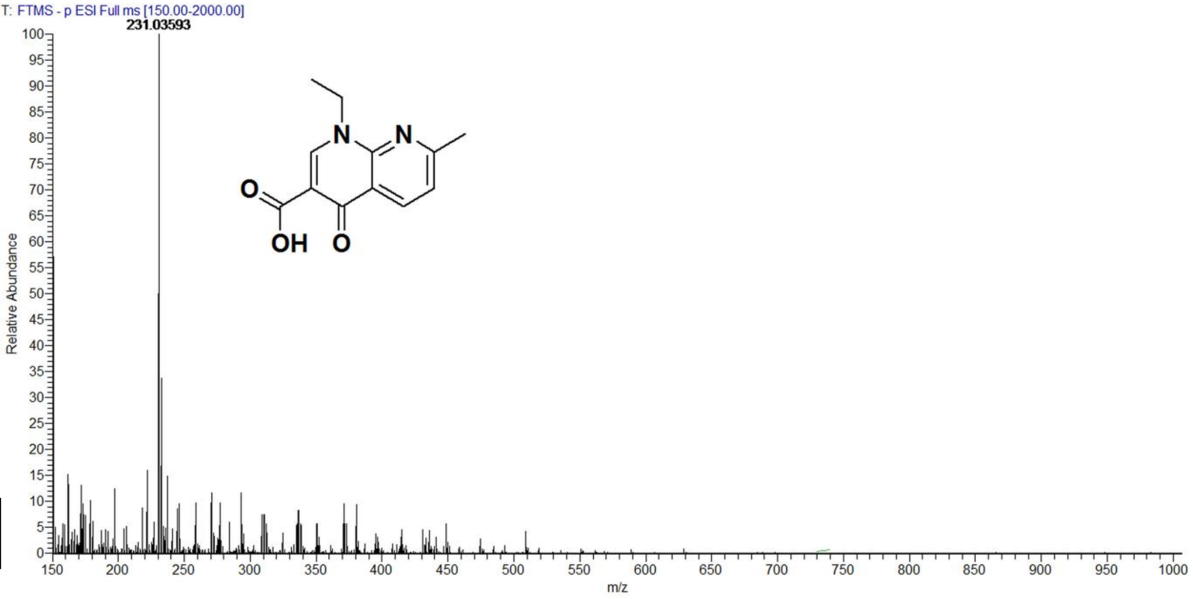
T: FTMS - p ESI Full ms [150.00-2000.00]
231.03593
N
N
O
OH
O
Relative Abundance
m/z

B

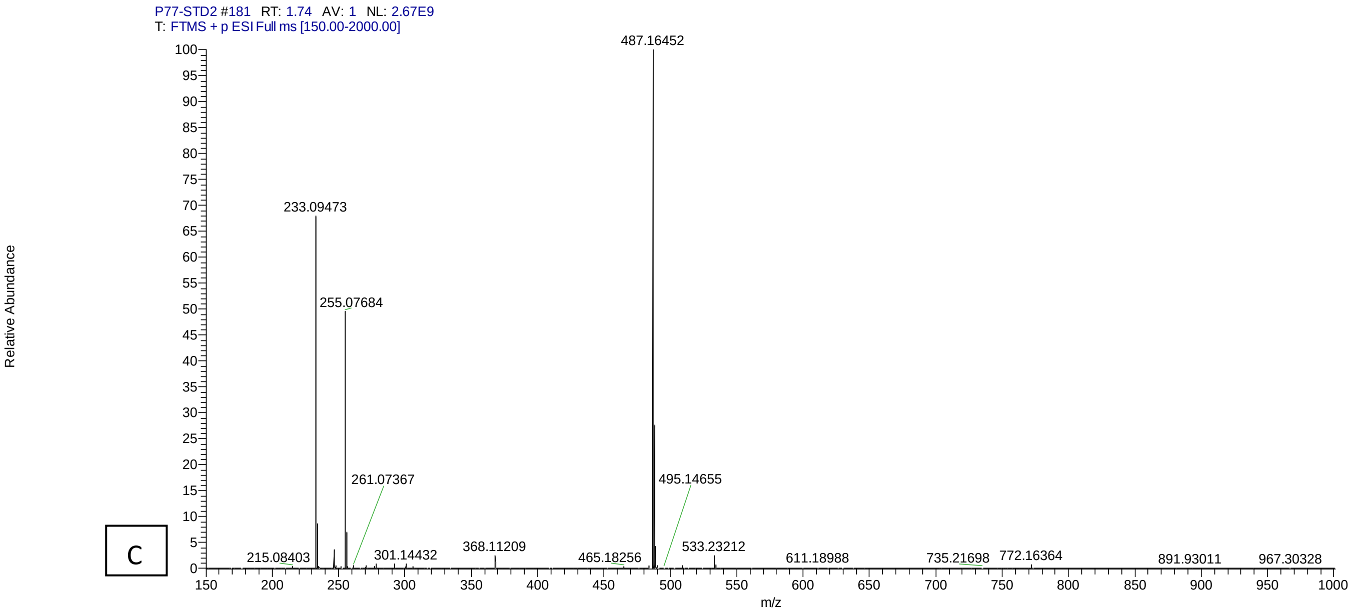

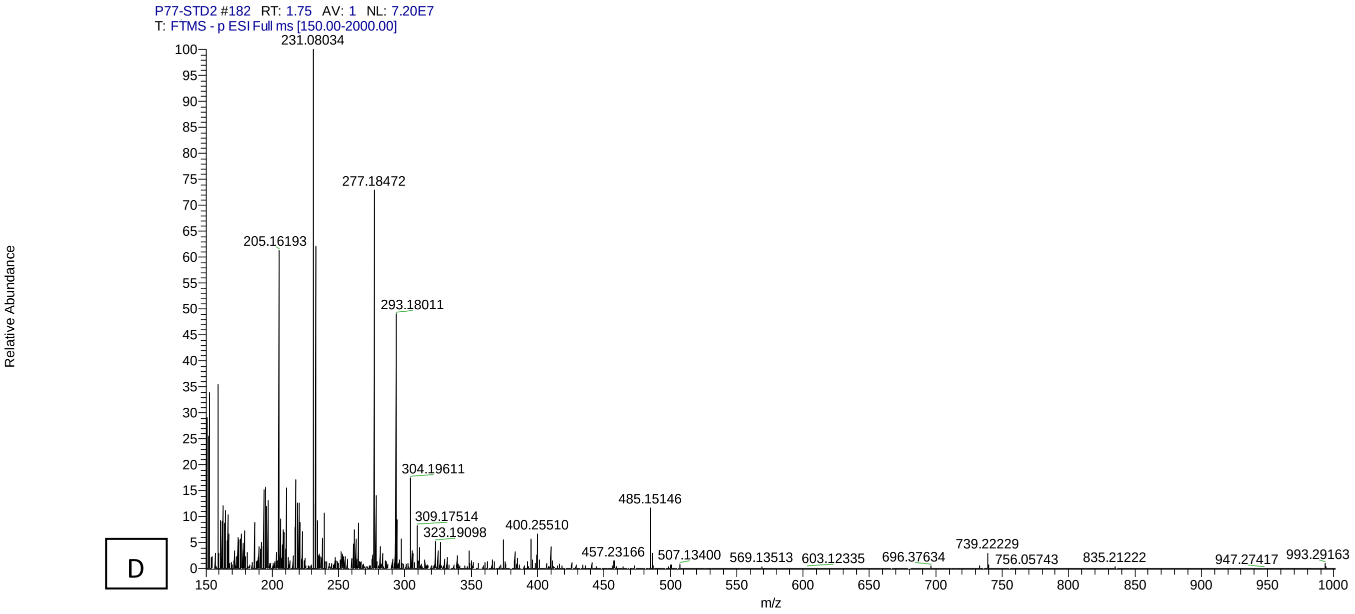

Additional file 6: Figure S4 Mass spectra of (A) nalidixic acid (B) flumequine present in PWS52 extract, (C) standard nalidixic acid (m/z : 233.09) in positive mode and (D) standard nalidixic acid (m/z: 231.08) in negative mode.
